# Double‐Stranded DNA Reduces dsRNA Degradation in the Saliva and Significantly Enhanced RNAi‐Mediated Gene Silencing in *Halyomorpha halys*

**DOI:** 10.1002/adbi.202400698

**Published:** 2025-08-17

**Authors:** Venkata Partha Sarathi Amineni, Georg Petschenka, Aline Koch

**Affiliations:** ^1^ Institute of Phytomedicine Department of Applied Entomology University of Hohenheim Otto‐Sander‐Straße 5 70599 Stuttgart Baden‐Württemberg Germany; ^2^ Institute of Cell Biology and Plant Biochemistry Department of Plant RNA Transport University of Regensburg Universitätsstraße 31 93053 Regensburg Bayern Germany

**Keywords:** DNA, dsRNA formulation, *Halyomorpha halys*, ribonucleases, RNAi

## Abstract

The invasive pest *Halyomorpha halys* (Hemiptera: Pentatomidae) poses a significant threat to agriculture and requires control methods beyond chemical pesticides. This study investigates RNA interference (RNAi) as a targeted gene silencing approach to manage *H. halys* populations. However, RNAi efficacy varies between insect orders, including hemipterans, due to factors such as the rapid degradation of double‐stranded RNA (dsRNA) by a DNA/RNA non‐specific nuclease (HhNSE) present in the saliva of *H. halys*. Notably, this study proves that double‐stranded DNA (dsDNA) can stabilise dsRNA in saliva, probably by competitively inhibiting HhNSE, which is highly expressed in salivary glands. In vivo tests targeting the *clathrin heavy chain* gene (*HhCHC*) demonstrate that a mixture of dsRNA‐CHC and dsDNA result in enhanced gene silencing when fed to *H. halys*, compared to dsRNA alone. While dsRNA‐CHC injection causes almost complete mortality, the dsDNA formulation do not significantly increase mortality when fed together with dsRNA‐CHC. These findings highlight the need to further investigate factors beyond nucleases such as dsRNA uptake and release mechanisms in the insect gut. Nevertheless, this study provides promising insights for improving RNAi delivery in *H. halys*, and perhaps other pests with such nucleases, in support of sustainable pest management solutions.

## Graphical abstract

The invasive pest *Halyomorpha halys* causes significant crop losses requiring innovative pest control strategies such as RNA interference (RNAi). However, oral RNAi is largely ineffective in *H. halys*. This study shows that double‐stranded DNA (dsDNA) stabilizes dsRNA in saliva by inhibiting a highly expressed nuclease. Feeding assays reveal enhanced gene silencing with dsDNA‐dsRNA mixtures, providing insights to improve RNAi‐based pest management.

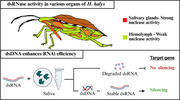

## 1 Introduction

The global demand for effective, species‐specific (i.e., selective), and sustainable alternatives to conventional chemical pesticides in agriculture is growing.^[^ ^1^ ^]^ RNA interference (RNAi) is a mechanism of gene regulation where short, non‐coding RNA molecules suppress the expression of complementary target genes (gene silencing). RNAi evolved as a natural defense against viruses in plants,^[^ ^2^ ^]^ mammals,^[^ ^3^, ^4^ ^]^ and insects.^[^ ^5^ ^]^ Modern RNAi‐based plant protection technologies utilize molecular mechanisms of post‐transcriptional gene silencing, which are initiated by Dicer, an RNase III‐like enzyme, breaking down a long double‐stranded (ds) RNA precursor into 21 to 24 nucleotide (nt) small interfering (si)RNA duplexes.^[^ ^6^ ^]^ Post‐transcriptional gene silencing occurs in the cytoplasm of the cell, where the siRNAs are incorporated into an RNA‐induced silencing complex (RISC)^[^ ^7^ ^]^ that contains an Argonaute protein with an RNA‐binding domain and endonucleolytic activity. In an ATP‐dependent reaction, siRNA is separated by RISC, resulting in a sense and an antisense strand. The sense strand, which is identical to the cell's endogenous mRNA, is degraded due to thermodynamic asymmetries while the antisense strand remains bound to the RISC and targets complementary mRNA transcripts for degradation.^[^ ^8^ ^]^

The efficiency of RNAi‐based control of insect pests varies drastically among insect orders (for an overview, see^[^ ^9^ ^]^). For example, 100% mortality were reached in the inbred German laboratory D01 strain of *Leptinotarsa decemlineata* (Coleoptera; Chrysomelidae) when fed with 30 ng dsRNA‐actin per single second instar larva on a 2 cm diameter leaf disc.^[^ ^10^ ^]^ In contrast, Lepidopterans such as *Spodoptera exigua* ^[^ ^11^ ^]^ and *Bombyx mori*,^[^ ^12^ ^]^ and Hemipterans, such as *Plautia stali*,^[^ ^13^ ^]^ *Euschistus heros*,^[^ ^14^ ^]^ and *Acyrthosiphon pisum*,^[^ ^15^ ^]^ were less sensitive to dsRNA when treated with relatively higher quantities of dsRNA per individual via feeding. Numerous factors likely contribute to this strong variation of RNAi efficiency including the degradation of dsRNA by nucleases in the saliva and gut of *Nezara viridula* ^[^ ^16^ ^]^ and the hemolymph of *Spodoptera frugiperda*.^[^ ^17^ ^]^ Additionally, one of the major impediments to RNAi efficiency in *S. frugiperda* is the accumulation of dsRNA in endosomes in cultured (Sf9), midgut and fat body cells.^[^ ^18^ ^]^ To bypass the challenge of dsRNA degradation by nucleases and other physiological barriers, protective formulations need to be developed which facilitate the delivery of dsRNA into target insect cells for inducing a proper post‐transcriptional gene silencing response.

*Halyomorpha halys* (Stål, 1855) (Hemiptera, Pentatomidae), the brown marmorated stink bug, is a pest invasive to Europe and the Americas. *H. halys* is a hemimetabolous insect that goes through five nymphal stages before becoming an adult.^[^ ^19^ ^]^ Due to its wide host range including fruits like apples, peaches, nectarines, pears, grapes, raspberries, and hazelnuts and crops like tomatoes, peppers, sweet corn, and soybeans and high reproductive output, it is responsible for severe economic losses in agri‐ and horticulture.^[^ ^20^ ^]^ In 2011, the first reports of *H. halys* emerged in both northern and southern regions of Germany. Within 4 years, multiple sightings were documented in the southern state of Baden‐Württemberg.^[^ ^21^ ^]^ Although *H. halys* was classified as an insect of special concern in Germany, it has not yet caused significant economic damage compared to other regions such as the United States and Italy.^[^ ^21^ ^]^ In Italy,^[^ ^22^ ^]^ for example, *H. halys* was first reported in 2012 and resulted in over EUR 588 million damage in apple production in the year 2019.^[^ ^23^ ^]^ To date, broad‐range chemical insecticides such as carbamates, neonicotinoids and pyrethroids etc., are the only effective control method for *H. halys* but pose environmental risks.^[^ ^24^, ^25^, ^26^ ^]^ Due to its high potential for selectivity, RNAi represents a promising method for sustainable *H. halys* management.^[^ ^27^, ^28^, ^29^ ^]^ However, high nuclease activity in the saliva and midgut extracts of adult *H. halys* ^[^ ^29^, ^30^ ^]^ might limit the delivery of dsRNA and thus, the efficiency of target gene silencing.

The DNA/RNA non‐specific nucleases (NSE) show a broad substrate affinity and digest dsRNA, dsDNA, single stranded (ss)RNA, ssDNA, and RNA/DNA hybrids.^[^ ^31^, ^32^ ^]^ Because NSEs degrade full length dsRNA efficiently, they are also referred to as dsRNases.^[^ ^33^ ^]^ An NSE was first identified in the midgut homogenate and liquid phase of the midgut of *B. mori* larvae,^[^ ^12^, ^34^ ^]^ and later studies revealed the strong expression of *NSE*s in a variety of insects^[^ ^17^, ^35^, ^36^, ^37^ ^]^ degrading dsRNA in the oral cavity, gut lumen and hemolymph before reaching the target cells. This ultimately reduces the efficiency of target gene silencing.

The clathrin protein plays a crucial role in the uptake of nutrients through the endocytosis pathway in eukaryotic cells.^[^ ^38^, ^39^ ^]^ Triskelion structured clathrin consists of three 190 kDa heavy chains and a single light chain (≈25 kDa), which connect at their C‐terminal ends to constitute the fundamental clathrin subunit.^[^ ^40^ ^]^ Knocking down the *clathrin heavy chain* (*CHC*) gene has been shown to result in significant mortality across various insect groups.^[^ ^41^, ^42^ ^]^

In this study, we aimed to evaluate a potential solution for protecting dsRNA from nuclease degradation when administered orally, which could further optimize the efficiency of RNAi in *H. halys* and potentially other insects. First, we evaluated the stability of dsRNA in saliva, salivary gland extracts, and hemolymph of *H. halys* across different developmental instars. To determine whether dsRNA degradation could be mediated by nucleases, we quantified the relative expression of ribonuclease genes, specifically DNA/RNA *non‐specific nuclease* (*HhNSE*), *exoribonuclease‐1* (*HhEri‐1*), and *small RNA degrading nuclease‐1* (*HhSDN‐1*), in the salivary glands of *H. halys* using RT‐PCR. Furthermore, we explored the capacity of both PCR amplified and ultrapure salmon‐sperm dsDNAs to act as a competitive inhibitor of HhNSEs and subsequently reduce dsRNA degradation in *H. halys* saliva through an ex vivo dsRNA degradation assay. Finally, we performed in vivo assays by orally administering dsRNA‐CHC targeting *HhCHC* (XM_01 443 1604.1), combined with dsDNA, to examine the efficacy of this formulation in enhancing oral RNAi efficiency. Our results suggest that formulating dsRNA with dsDNA can bypass the severe dsRNase activity in the saliva, thereby enhancing oral RNAi efficiency in *H. halys*, but without a significant effect on mortality.

## 2 Results

### 2.1 Ex Vivo dsRNA Stability in Saliva, Salivary Gland Extract, and Hemolymph of *H. halys*

#### 2.1.1 Saliva

**Figure 1:**
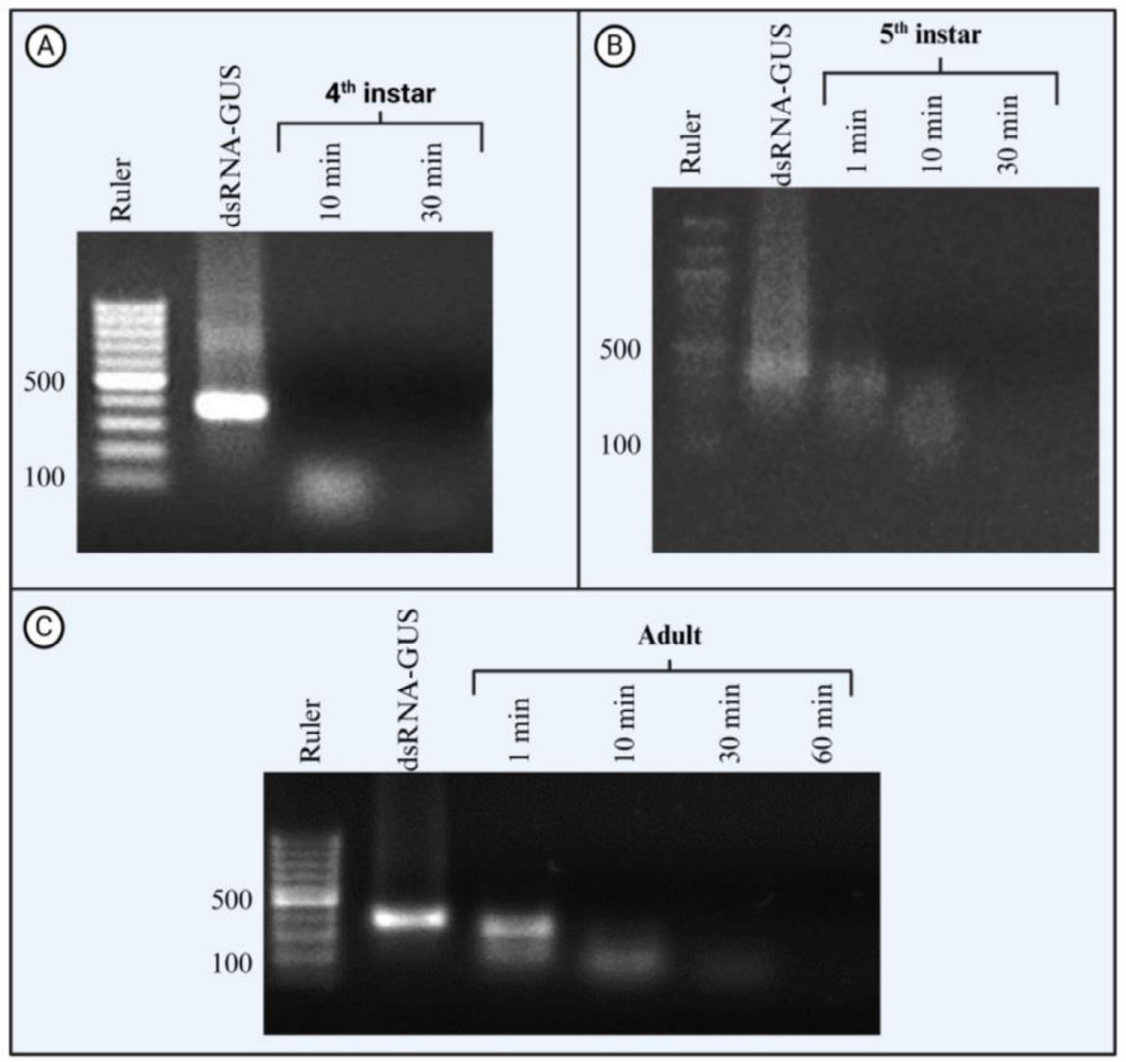
The degradation of dsRNA in saliva is strong in all tested developmental stages of *H. halys*. Ex vivo dsRNA degradation assay of dsRNA‐GUS incubated with the saliva of *H. halys*. Saliva (2 µL) was collected from *H. halys* fourth instar nymphs (A), fifth instar nymphs (B), and adults (C) incubated with 2 µg of dsRNA‐GUS at 25 °C for up to 30–60 min, and 1% sodium dodecyl sulfate (SDS) was added to the samples to stop the nuclease reaction. The samples were run on a 1% agarose gel, and the experiment was replicated twice for each growth stage except for fourth instar (*n* = 1). In all gels, DNA ladder is used to cross‐check the length of dsRNA strand and quality of the gel staining.

Incubation of dsRNA‐GUS with undiluted *H. halys* saliva resulted in severe degradation of dsRNA‐GUS (**Figure** **1**). The degree of dsRNA degradation was similar for the fourth, fifth, and adult stages of *H. halys* (Figure 1). After only 1 min of incubation with saliva collected from each of the three stages, a diffuse band with a large amount of smear was already detected on the gel. After 10 min, the dsRNA‐GUS signal disappeared from the gel, indicating that the dsRNA was completely degraded.

#### 2.1.2 Salivary Glands

**Figure 2:**
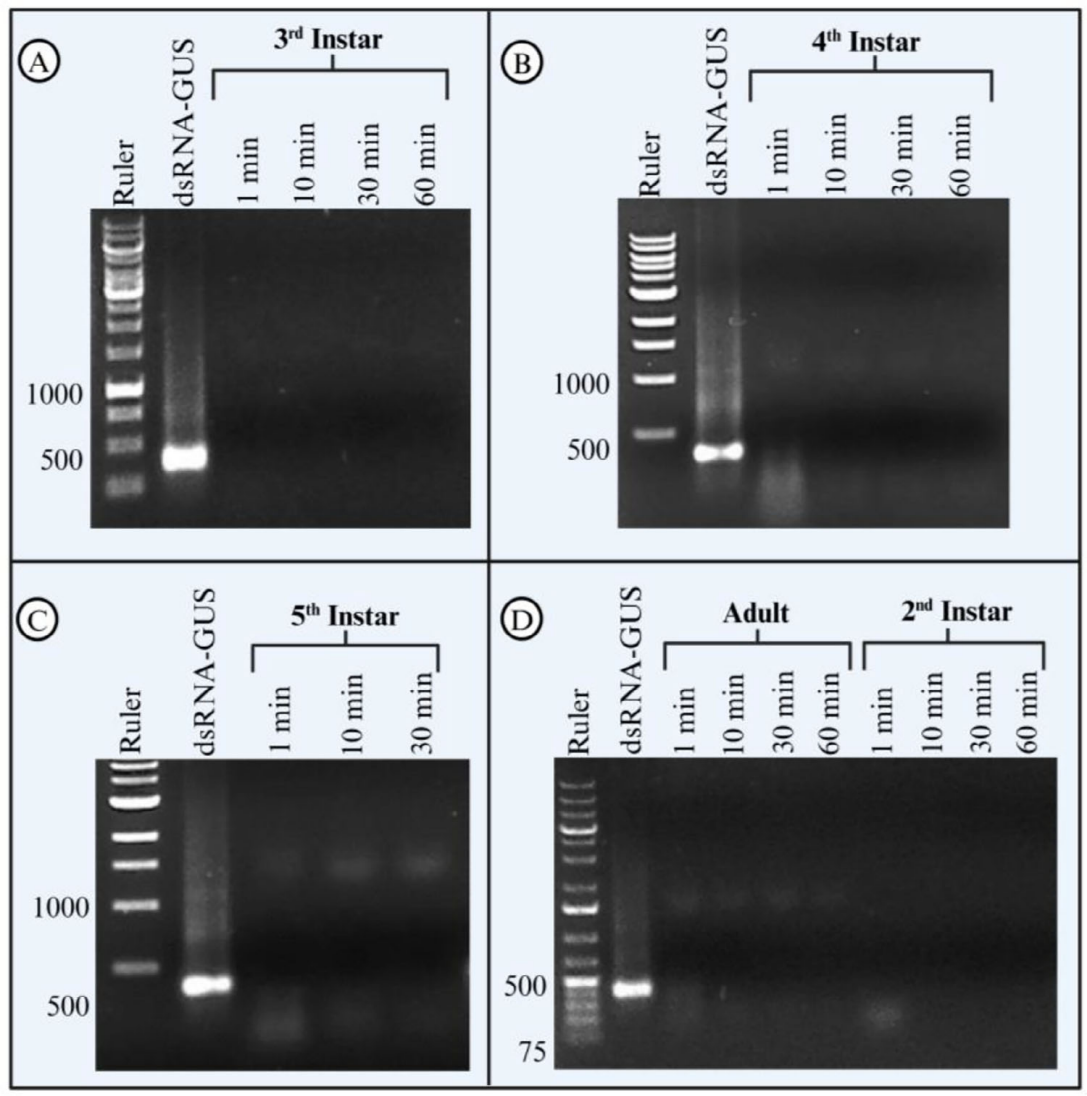
Salivary gland extracts of *H. halys* rapidly degrade dsRNA within 1 min across all developmental stages. Ex vivo dsRNA degradation assay of dsRNA‐GUS incubated with *H. halys* salivary gland extract. Salivary gland extract (2 µL) was extracted from *H. halys* third instar nymphs (A), fourth instar nymphs (B), fifth instar nymphs (C), and adults and second instar nymphs (D) and incubated with 2 µg of dsRNA‐GUS for up to 60 min at 25 °C, and 1% SDS was added to the samples to stop the nuclease reaction. The samples were run on a 1% agarose gel and the experiment was replicated twice. DNA ladder was used in all gels.

In addition to saliva collected from live insects, we analysed the dsRNA degradation rate in salivary gland extracts from all *H. halys* growth stages, except the first instar larvae. We found that 2 µL of salivary gland extract was sufficient to digest 2 µg of 100 ng µL^−1^ dsRNA‐GUS in less than 1 min (*F*_1, 78_ = 496.95, *p* < 0.001) (**Figure** **2**) when compared to dsRNA‐GUS incubated with water. No significant difference was found between different time points (*F*_4, 78_ = 0.23, *p* = 0.918) as dsRNA‐GUS was already completely degraded within 1 min. Salivary gland extracts from all growth stages showed similar degradation activity (Figure 2).

#### 2.1.3 Hemolymph

**Figure 3:**
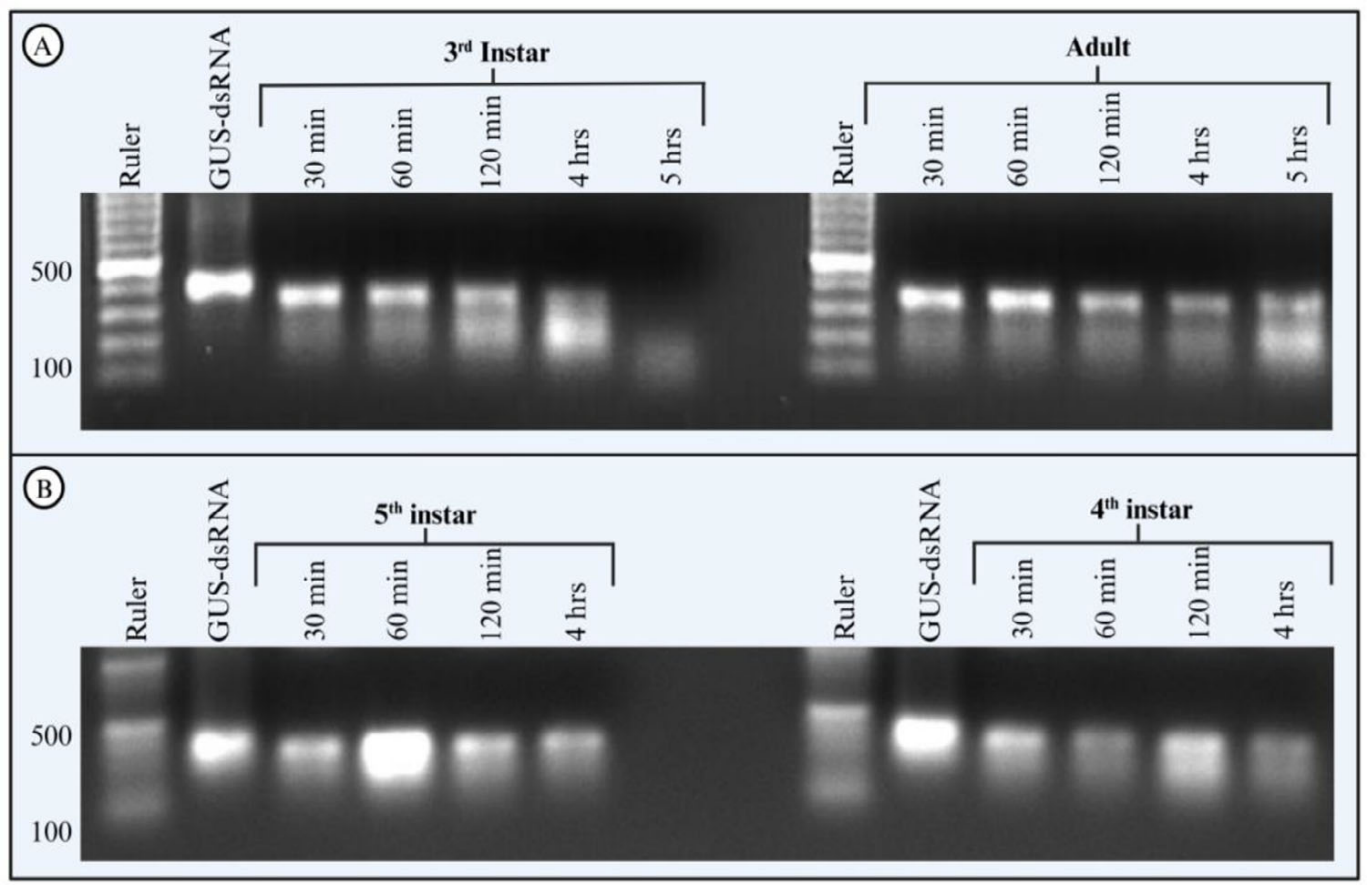
Hemolymph of *H. halys* degrades dsRNA more slowly than saliva or salivary gland extracts, with degradation rates varying slightly with developmental stage. Ex vivo dsRNA degradation assay of dsRNA‐GUS incubated with *H. halys* hemolymph. Hemolymph (2 µL) from *H. halys* third instar nymphs and adults (A) and fifth instar nymphs and fourth instar nymphs (B) was incubated with 2 µg dsRNA‐GUS at 25 °C for up to 4–5 h, and 1% SDS was added to the samples to stop the nuclease reaction. The samples were run on a 1% agarose gel and the experiment was replicated twice.

The dsRNase activity was lower in hemolymph than in saliva or salivary gland extracts of *H. halys*, as dsRNA‐GUS remained intact for up to 2 h after ex vivo incubation (**Figure** **3**). Degradation of dsRNA began immediately after the start of incubation, but the degradation rate was lower compared to salivary nucleases (Figures 1 and 2). Complete degradation of dsRNA‐GUS occurred earlier when dsRNA‐GUS was treated with hemolymph isolated from third instar nymphs (Figure 3A), where the dsRNA signal clearly started to disappear from the gel after 4 h (Figure 3A). In contrast, the dsRNA‐GUS from the fourth, fifth instar and adult samples remained somewhat stable, while a smear in the gel indicated degradation products that were present as early as 30 min after incubation began (Figure 3). Statistical analyses resulted that there is significant variation in dsRNA stability between different growth stages (*F*_1, 61.7_ = 19.24, *p* < 0.001) as well as between the nuclease free water and hemolymph incubated samples (*F*_4, 61_ = 5.54, *p* < 0.001).

### 2.2 Comparison of dsRNase Expression in Salivary Glands

**Figure 4:**
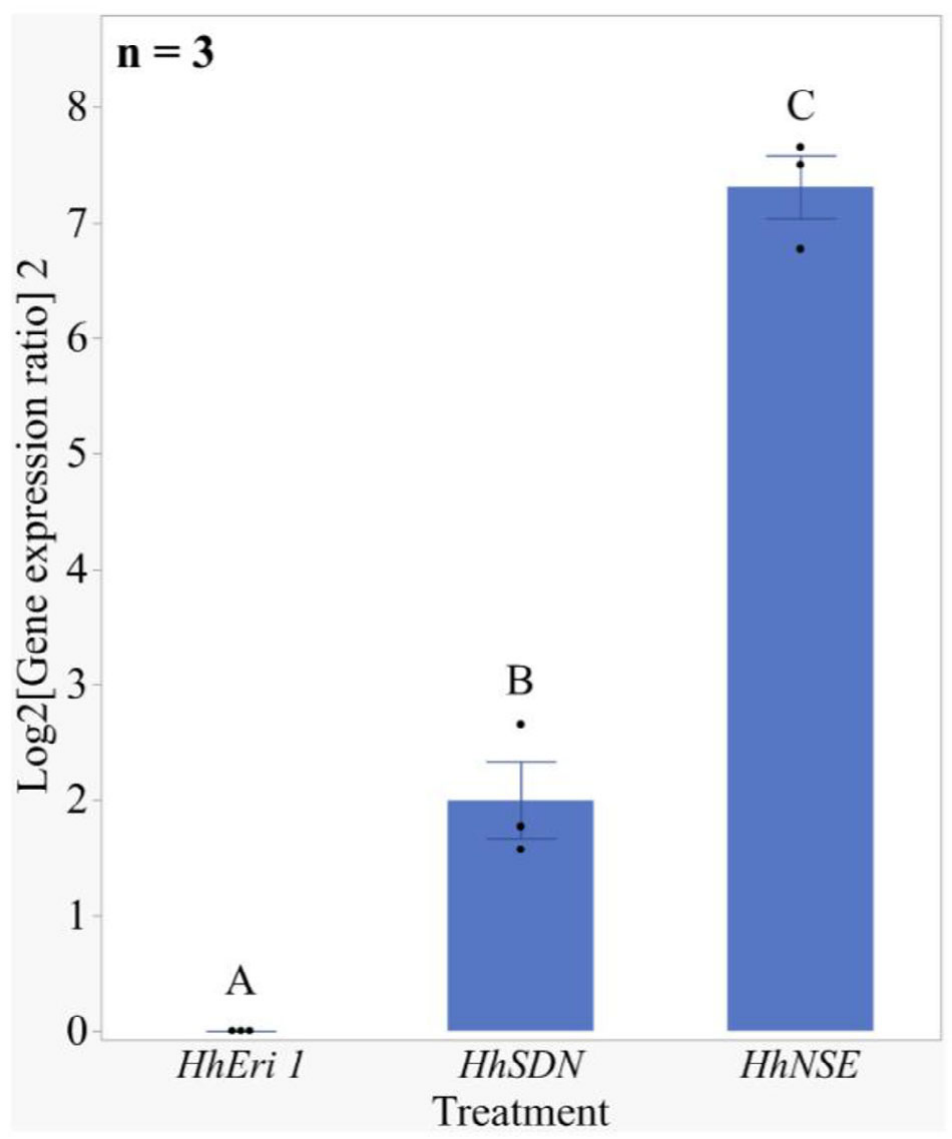
*HhNSE* is the predominant dsRNA‐degrading nuclease in *H. halys* salivary glands. Relative expression levels of dsRNA degrading nucleases (*HhEri‐1, HhSDN‐1, HhNSE*) in *H. halys* salivary glands determined by RT‐PCR. Total RNA from 18–20 pooled salivary glands (from ≈10 untreated *H. halys* adults) was isolated and considered as one biological sample (n). A total of three independent biological replicates (n = 3) were evaluated, with each replicate subjected to two technical replicates. The Pfaffl method was used to adjust the threshold cycle (Ct) values, taking into account primer efficiency variations, and to calculate relative gene expression ratios using *HhEri‐1* as a calibrator gene. Statistical comparison of gene expression variability was conducted by ANOVA, and treatments with different letters indicate significant differences (Tukey‐HSD, *p* < 0.01).

The expression levels of *HhNSE*, *HhEri‐1* and *HhSDN‐1* in the salivary glands of adult *H. halys* were analysed by RT‐PCR, as detailed in Table S3, Supporting Information. It was found that *HhNSE* showed a significantly higher expression in the salivary glands compared to the other nucleases investigated, in the order *HhNSE* > *HhSDN*‐1 > *HhEri‐1*, as shown in **Figure** **4**. Pairwise comparisons with Tukey‐HSD revealed significant differences; *HhNSE* versus *HhEri‐1* (Difference = 7.31, SE = 0.35, *p* < 0.0001), *HhNSE* versus *HhSDN‐1* (Difference = 5.31, SE = 0.35, *p* < 0.0001), and *HhEri‐1* versus *HhSDN‐1* (Difference = 2.0, SE = 0.35, p = 0.003). A detailed analysis and results are provided in Table S3 & SI_2.pdf, Supporting Information.

### 2.3 dsDNA Competitively Inhibits dsRNA Degradation by Salivary Nucleases in *H. halys*

**Figure 5:**
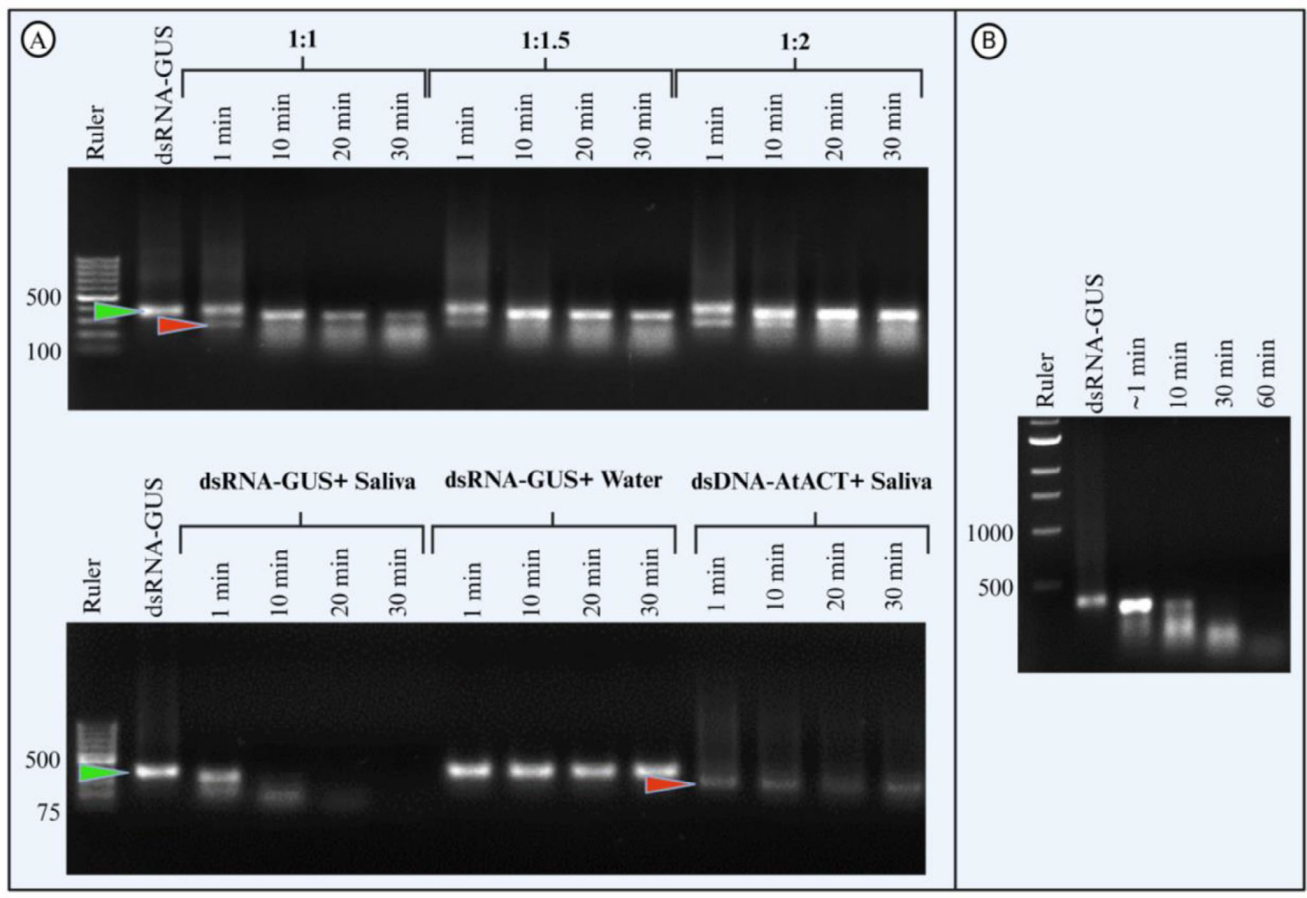
dsDNA competitively inhibits dsRNA degradation by *H. halys* salivary nucleases. dsRNA‐GUS (2 µg; green arrowhead) incubated with adult *H. halys* saliva in the presence of different concentrations of 246 bp dsDNA‐AtACT from *A. thaliana* (red arrowhead)(A). Aliquots collected at 1, 10, 20, and 30 min after incubation start. Reaction stopped with 1% SDS. dsRNA‐GUS incubated in 2 µL of adult *H. halys* saliva at varying dsRNA:dsDNA ratios (e.g., 1:2). Double concentration of dsRNA‐GUS (4 µg) incubated with 2 µL of undiluted saliva (B) from the same batch as in (A). Reaction mixtures incubated at 25 °C, with aliquots taken at increasing time points up to 60 min and stopped with 1% SDS. All samples visualized on a 1% agarose gel with 1 × TAE buffer to assess stability of dsRNA‐GUS and dsDNA‐AtACT.

In *H. halys* salivary glands, *HhNSE* was highly expressed compared to the other known dsRNases. Therefore, *HhNSE* may be the dominant enzyme responsible for dsRNA degradation in *H. halys* saliva. The broad substrate affinity of *HhNSEs* for nucleic acids provided a potential opportunity to protect dsRNA by competitively inhibiting *HhNSEs* with dsDNA. Therefore, we investigated the stability of dsRNA when incubated with *H. halys* saliva with the addition of dsDNA. dsRNA‐GUS (2 µg) was incubated with 2 µL of undiluted adult *H. halys* saliva alone and with 2 µg, 3 µg, and 4 µg of 246 bp dsDNA‐*At*ACT separately. The degradation rate of dsRNA‐GUS decreased with increasing concentration of 246 bp dsDNA‐AtACT (**Figure** **5**A). A distinct band of dsRNA‐GUS was detected at 30 min when dsRNA‐GUS was co‐incubated with dsDNA‐AtACT and saliva samples at all dsRNA:dsDNA ratios (Figure 5A). In contrast, the 2 µL of saliva from the same stock completely degraded the 4 µg of dsRNA‐GUS within 30 min of incubation when no dsDNA was added (Figure 5B).

**Figure 6:**
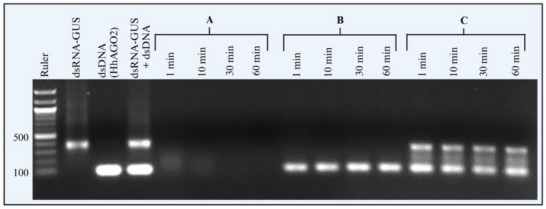
The shorter 102 bp dsDNA‐HhAGO2 also protects dsRNA from degradation by *H. halys* salivary nucleases. Testing the stability of dsRNA‐GUS with saliva by co‐incubation with 102 bp dsDNA. dsRNA‐GUS (2 µg) was incubated with 2 µL of *H. halys* saliva (A). 102 bp dsDNA‐HhAGO2 (2 µg) incubated with 2 µL of *H. halys* saliva (B). dsRNA‐GUS (2 µg) with an equal amount of 102 bp dsDNA‐HhAGO2 was incubated with 2 µL of *H. halys* saliva (C). After incubation, samples were collected at 1, 10, 30, and 60 min and 1% SDS was added to stop the reaction. The samples were visualized on a 1% agarose gel with 1xTAE buffer to evaluate the stability of dsRNA‐GUS and dsDNA‐HhAGO2.

In addition to the 246 bp dsDNA‐AtACT from *A. thaliana*, we tested whether the shorter 102 bp dsDNA‐*Hh*AGO2 fragment from *H. halys AGO‐2* also mediates dsRNA protection from *Hh*NSEs and carried out the degradation experiment under the same experimental conditions (**Figure** **6**). We tested the stability of 2 µg of dsRNA‐GUS when incubated with 2 µL of adult *H. halys* saliva and 2 µg of 102 bp long dsDNA‐HhAGO2. Consistent with the results for the 246 bp dsDNA‐AtACT, the 102 bp dsDNA‐HhAGO2 also reduced the dsRNA‐GUS degradation when added to the reaction at equal concentrations (1:1). dsRNA‐GUS remained stable up to 60 min after incubation with saliva (Figure 6C) and showed a distinct band on the gel, but in the identical reaction without dsDNA‐HhAGO2, dsRNA‐GUS was completely degraded within 1 min of treatment with *H. halys* saliva (Figure 6A).

Taken together, these results demonstrate the potential of dsDNA as a competitive substrate for dsRNA‐ degrading nucleases in *H. halys* saliva. We suggest that dsDNA significantly saturates *Hh*NSE, limiting the availability of *Hh*NSEs to degrade dsRNA‐GUS. The combined analysis of quantified amounts of dsRNA on gel images at a 1:1 ratio of dsRNA‐GUS to dsDNA‐(AtACT (n = 2)/HhAGO2 (n = 2)), using ImageJ and SAS 9.0, revealed a significant increase in dsRNA‐GUS stability compared to the treatment without dsDNA (*F*_2,25.1_ = 26.86, *p <* 0.001) (Figures S4–S6, Supporting Information).

**Figure 7:**
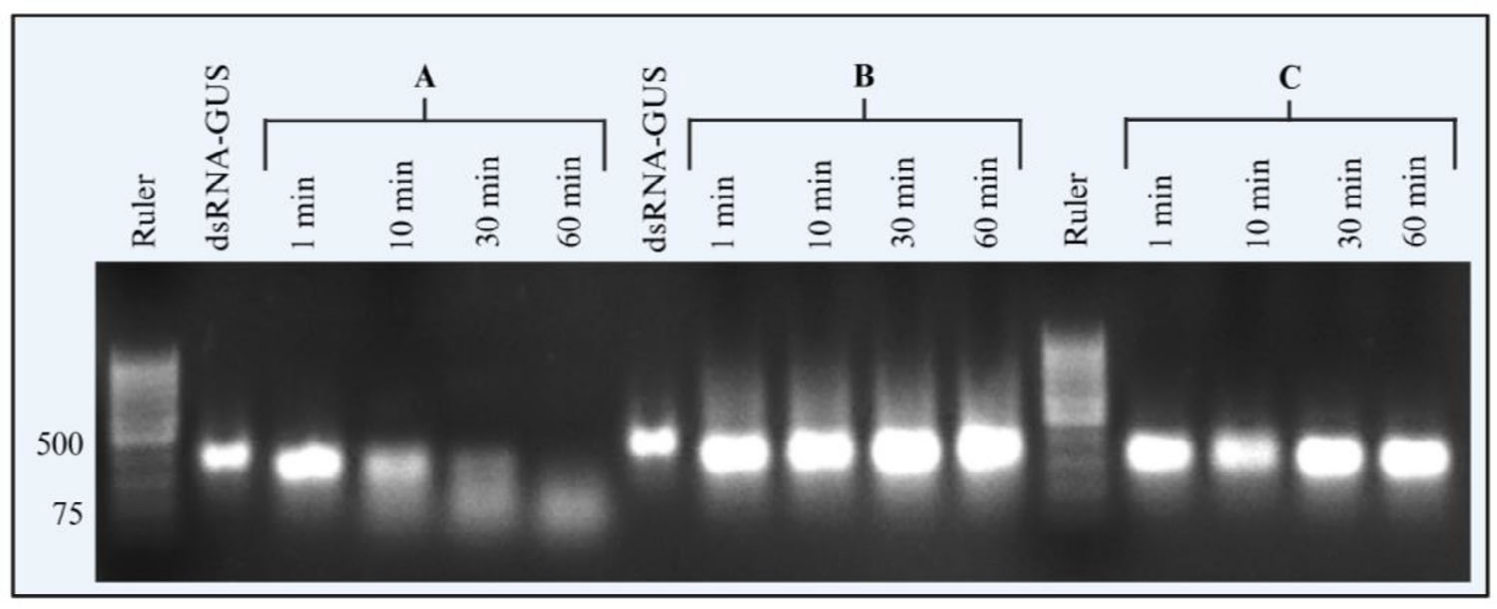
Ultra‐Pure Salmon sperm DNA (dsDNA‐S) enhances dsRNA stability in *H. halys* saliva. dsRNA‐GUS (2 µg) was incubated with 2 µL of *H. halys* saliva (A). dsRNA‐GUS (2 µg) with an equal amount of dsDNA‐S was incubated with 2 µL of *H. halys* saliva (B). dsRNA‐GUS (2 µg) was incubated with 2 µL of Nuclease free water (C). After incubation, samples were collected at 1, 10, 30, and 60 min and 1% SDS was added to stop the reaction. The samples were visualized on a 1% agarose gel with 1xTAE buffer to evaluate the stability of dsRNA‐GUS and dsDNA‐S (*n* = 2).

Ultra‐pure crude dsDNA‐S also enhanced stability of dsRNA when co‐incubated with saliva, as illustrated in **Figure** **7**. As the results were clearly indicative of enhanced dsRNA stability, this experiment was only replicated twice. Subsequently, dsDNA‐S was used as a co‐formulant in dsRNA feeding experiments.

### 2.4 Use of Salmon Sperm DNA (dsDNA‐S) as a Formulation Agent to Improve Oral dsRNA Efficacy

**Figure 8:**
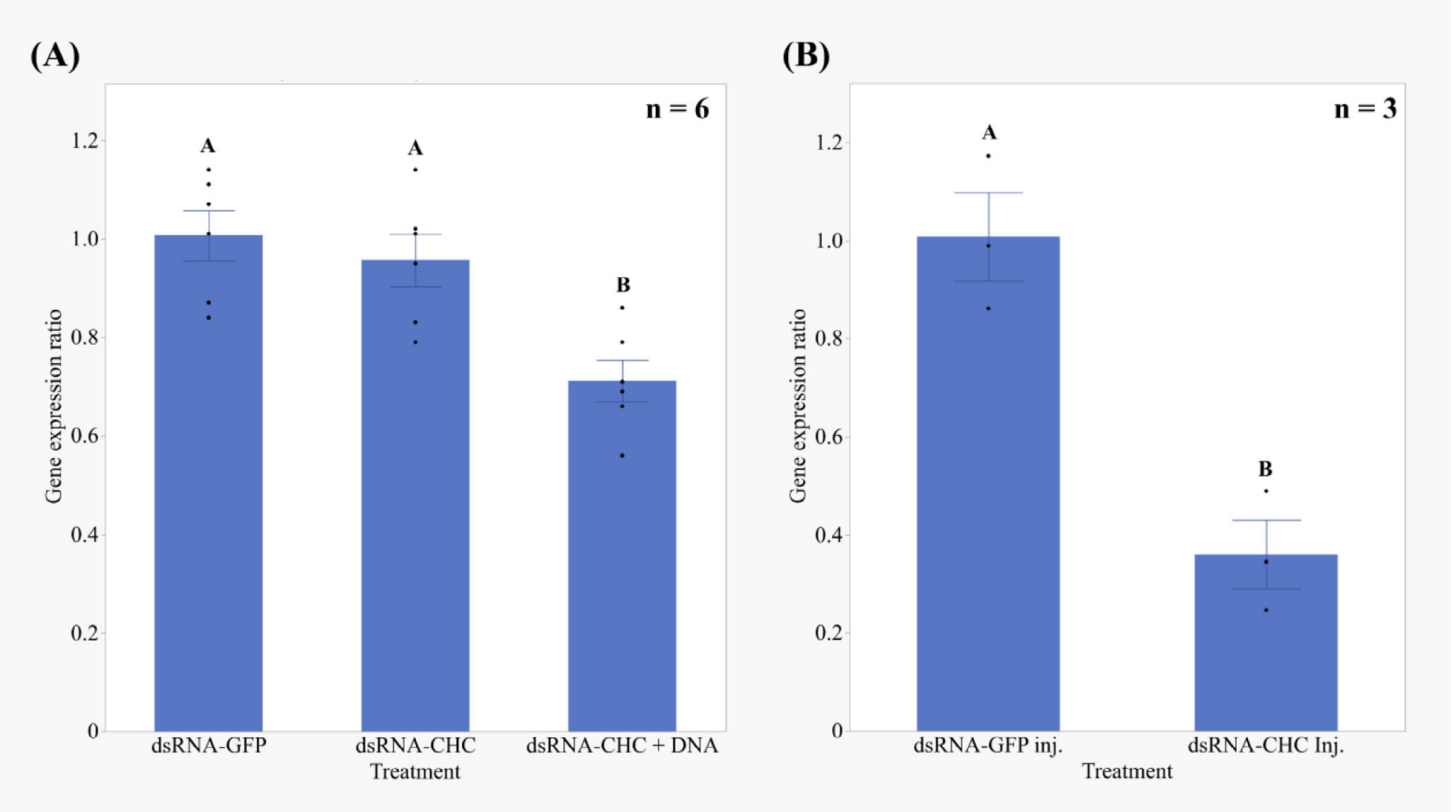
Application of dsDNA‐S enhances oral RNAi efficacy in *H. halys*. Efficiency of target gene knockdown was evaluated by RT‐PCR in dsRNA‐fed (A) and injected (B) second instar *H. halys* 72 h post‐treatment. Nymphs were fed 20 µg dsRNA‐CHC, 20 µg dsRNA‐GFP, or a combination of 20 µg dsRNA‐CHC and 20 µg dsDNA‐S and injected with 100 ng dsRNA per nymph. Total RNA was converted to cDNA, and mRNA levels were normalized to 18S rRNA. Mean ± SE of relative *HhCHC* mRNA levels are shown. Statistical comparisons were made using a standard least squares model with a Tukey‐HSD test for multiple comparisons. Treatments with different letters indicate significant differences (*p <* 0.008).

**Figure 9:**
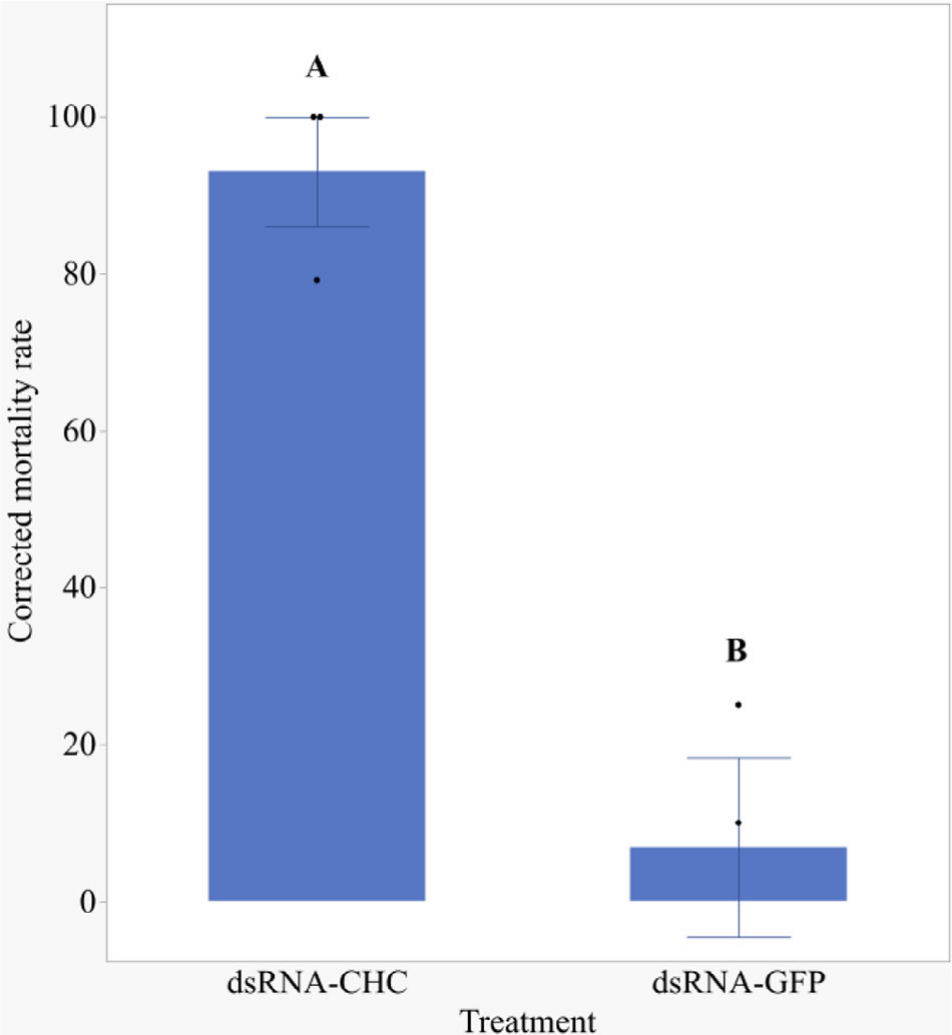
Injection of dsRNA‐CHC significantly increases mortality in *H. halys* nymphs. Corrected mortality rates of second instar *H. halys* nymphs injected with dsRNA‐CHC, and dsRNA‐GFP. In total, three experiments were performed, with sample sizes ranging from 14 to 18 individuals per treatment. Mortality was assessed over 13 days and cumulative mortality rates were corrected for the mortalities observed in the NF water treatment using Schneider‐Orelli's formula. Data were analysed by ANOVA followed by Tukey‐HSD post‐hoc test. Means ± SE shown represent the average of the mean cumulative mortality rates across the three independent experiments (n = 3). Treatments with different letters indicate significant differences (Tukey‐HSD, *p* < 0.001).

To test whether the formulation of dsRNA with DNA increases the efficacy of dsRNA‐mediated RNAi in live *H. halys*, we examined the effect of orally administered dsRNA‐CHC, combined with dsDNA‐S, on the silencing of the *HhCHC* gene (**Figure** **8**A). Feeding was confirmed by coloured feeding spots (i.e., salivary sheaths stained with food dye) on parafilm sachets (Figure S1, Supporting Information). We performed a dsRNA‐CHC injection assay as a positive control to assess whether feeding our formulated dsRNA achieved comparable gene silencing, given that injection causes high mortality (see **Figure** **9**).

While *HhCHC* expression levels in insects orally treated with dsRNA‐CHC did not differ from insects fed with dsRNA‐GFP (p = 0.75), *HhCHC* expression was significantly reduced when dsDNA‐S was co‐administered with dsRNA‐CHC (p = 0.008, dsRNA‐CHC + dsDNA‐S versus dsRNA‐CHC; p = 0.001, dsRNA‐CHC + dsDNA‐S versus dsRNA‐GFP). However, direct injection of dsRNA‐CHC resulted in an even more pronounced reduction of *HhCHC* expression, approximately sixfold compared to dsRNA‐GFP injection (*p <* 0.004) (Figure 8B). A detailed statistical analysis report is provided in Supporting Information “files SI_3.pdf” (injection assay) and “SI_4.pdf” (feeding assay).

### 2.5 dsRNA‐CHC Injection Significantly Increases Mortality in *H. halys* Compared to Controls

To assess the impact of dsRNA‐CHC on the survival of *H. halys*, we selected second‐stage nymphs and injected 100 nL (1 µg µL^−1^ concentration) of dsRNA per insect. Survival data were recorded over a 13‐day period following injection and cumulative mortality rates were corrected using the Schneider‐Orelli formula using the mortality rate in the NF treatment as a baseline.

We observed high mortality upon the injection of dsRNA‐CHC close to 100 percent when compared to the injection with dsRNA‐GFP (corrected mortality means dsRNA‐CHC: 93.056% ± 7.72 SE, dsRNA‐GFP: 6.9% ± 7.72 SE, Figure 9). Statistical analysis of our data clearly revealed that the injection of dsRNA‐CHC caused substantially higher mortality compared to the dsRNA‐GFP (ANOVA, Tukey post hoc test, *p <* 0.001). A detailed statistical analysis report can be found in the Supporting Information file, entitled “SI_5.pdf.”

### 2.6 Survival of *H. halys* Following Oral Ingestion of dsRNA‐CHC With and Without dsDNA

To evaluate the effect of adding dsDNA to dsRNA‐CHC on *H. halys* survival via oral delivery, we fed second‐stage nymphs 200 µL of dsRNA (100 ng µL^−1^) in groups of five. Survival was monitored over 14 days while they fed on fresh green bush beans post‐treatment. Please refer to the Supporting Information, which can be found as SI_6.pdf, for a detailed statistical analysis report.

No significant differences were observed across all treatments (GLM, χ^2^(6) = 6.6784, *p* = 0.3516), indicating that the oral delivery of dsRNA‐CHC did not result in increased mortality, irrespective of the addition of dsDNA (Figure S3, Supporting Information).

## 3 Discussion

RNA interference (RNAi) has emerged as a promising strategy for pest control, offering specificity and reduced environmental impact compared to traditional chemical pesticides. Our study addresses a major challenge in RNAi‐based insect pest control: the degradation of dsRNA by nucleases in the oral cavity. We found that both saliva and salivary gland extracts of *H. halys* rapidly degrade dsRNA in vitro, a finding that is consistent across all tested developmental stages of the pest. Furthermore, our data suggest that HhNSE is a key enzyme involved in this degradation process. Our main finding is that dsDNA acts as a competitive inhibitor to protect dsRNA from enzymatic degradation, significantly increasing its stability and the efficacy of gene silencing.

dsRNases impair the oral delivery efficiency of dsRNA in many insects, including *H. halys*, thereby reducing the effectiveness of RNAi‐based pest control strategies.^[^ ^9^, ^43^ ^]^ Notably, previous research documented significant dsRNA degradation in the saliva of adult *H. halys*.^[^ ^29^ ^]^ However, it remained uncertain whether this degradation extends to other developmental stages. Our demonstration of dsRNase activity in saliva, salivary gland extracts and hemolymph from different developmental stages of *H. halys* may support future strategies for effective control of this important insect pest.

Our ex vivo dsRNA stability assays in *H. halys* have shown that dsRNA exhibits prolonged stability in the hemolymph, exceeding 4 h, in contrast to its rapid degradation in saliva and salivary gland extracts at all growth stages tested (Figures 1 and 7). This observation is consistent with the other insects with functional RNAi mechanism when dsRNA injected directly into the hemolymph.^[^ ^44^, ^45^ ^]^ In such insects, dsRNA remains intact and functional when injected into the hemolymph, thereby ensuring dsRNA to remain active long enough to be taken up by cells and activate the RNAi mechanism, resulting in target gene silencing. The constancy of this pattern across different RNAi‐sensitive insect species highlights the significance of dsRNA stability in the efficacy of RNAi.

Conversely, the rapid destruction of dsRNA observed in *H. halys* saliva is consistent with findings in RNAi‐resistant insects.^[^ ^15^, ^17^, ^33^, ^46^, ^47^ ^]^ Salivary nucleases rapidly degrade dsRNA in these species, preventing the dsRNA from entering into the cells and silencing target genes, leading to ineffective oral‐route RNAi. For example, in *N. viridula* where severe nuclease activity was observed in saliva, silencing NSE improved oral RNAi efficacy to 65% mortality compared to 43.3% without NSE silencing when treated with dsRNA targeting the *alpha coatomer* gene.^[^ ^36^ ^]^ We found that *HhNSE* has significantly higher expression levels in salivary glands compared to other known insect dsRNase's such as *HhEri‐1* and *HhSDN‐1*,^[^ ^17^, ^48^, ^49^ ^]^ suggesting a predominant role of *HhNSE* in salivary dsRNA digestion. This is supported by literature data demonstrating that silencing of *NSE* in other species such as *S. frugiperda*,^[^ ^50^ ^]^ *N. viridula*,^[^ ^36^ ^]^ *Bemisia tabaci*,^[^ ^51^ ^]^ and *Tribolium castaneum* ^[^ ^52^ ^]^ enhances the efficacy of RNAi.

Given the observed broad substrate specificity of NSEs,^[^ ^31^, ^32^ ^]^ we hypothesised that dsDNA could act as a competitive inhibitor for salivary HhNSE and thereby mitigate dsRNA degradation in *H. halys*. Our results (Figures 6 and 7) clearly demonstrate that the addition of dsDNA to saliva samples significantly reduces dsRNA degradation, marking the first instance of using DNA as a competitive inhibitor against dsRNA‐degrading nucleases. It would be beneficial to further characterise the principle of competitive inhibition by producing and purifying the enzyme in order to test the efficiency of dsDNA in protecting dsRNA from HhNSE‐mediated degradation in the future.

The in vivo results demonstrate that the presence of dsDNA‐S not only enhances the stability of dsRNA in ex vivo conditions but also amplifies RNAi efficacy in vivo (Figure 8). This is indicated by the increased efficacy of target gene silencing achieved by ingesting both dsDNA‐S and dsRNA‐CHC together. Nevertheless, the efficacy of *HhCHC* silencing achieved by the oral delivery of dsDNA‐S in conjunction with dsRNA‐CHC is considerably lower to that observed following dsRNA‐CHC injection. The direct injection of dsRNA‐CHC into the hemocoel resulted in complete mortality within 13 days, a finding that is consistent with previous studies on other Pentatomidae, such as *N. viridula*.^[^ ^36^ ^]^ In contrast, the oral delivery of dsRNA‐CHC combined with dsDNA‐S resulted in lower gene silencing levels that did not induce mortality in *H. halys*. Nevertheless, the RT‐PCR results indicating a considerable enhancement in target gene silencing suggest that the incorporation of dsDNA as a co‐formulant to competitively inhibit NSE is a promising strategy. However, in order to optimise this approach, it is important to address the other possible biological barriers that impede the delivery of dsRNA.

The potential biological barriers to dsRNA delivery in *H. halys* can be broadly categorised as follows: (a) dsRNase activity in the oral tract, (b) a lack of dsRNA uptake and release in epithelial cells, (c) restricted functionality of the RNAi machinery, and (d) No or restricted intercellular transport of dsRNA. Our injection assays demonstrated that barriers (c) and (d) are not present, while barrier (a) can be overcome by our DNA formulation. Therefore, the remaining barrier (b) may have restricted the efficient oral delivery of dsRNA. For example, it could be tested whether the dsRNA uptake behaviour differs between the apical and basal side of midgut epithelial cells, specifically whether the apical side exposed to the midgut lumen would be impermeable while dsRNA would be able to enter midgut cells from the basal side. This hypothesis could be tested by quantifying gene silencing by RT‐PCR in epithelial cells following direct injection of dsRNA into the hemolymph.

The reason for the lower gene silencing observed with dsDNA plus dsRNA feeding in *H. halys* can be related to difficulties with dsRNA uptake into midgut cells. Beyond the endocytic dsRNA uptake mechanism in midgut cells, SID‐like proteins have been shown to play a crucial role in dsRNA uptake in several insects such as *L. decemlineata, Diabrotica virgifera virgifera*.^[^ ^53^, ^54^ ^]^ In the hemipteran insect *Nilaparvata lugens*, a SID‐like protein was shown to be involved in dsRNA uptake and to increase the efficiency of systemic RNAi.^[^ ^55^ ^]^ In contrast, no significant role of SID‐like proteins in dsRNA uptake was observed in *T. castaneum* and *Bactrocera dorsalis*.^[^ ^56^, ^57^ ^]^ Suggesting that SID‐like proteins are not always the critical mediator for systemic RNAi in insects. However, as Sparks M et al. 2014^[^ ^58^ ^]^ have indicated, SID or SID‐like protein is not present in the *H. halys* transcriptome, which adds further evidence of the potential difficulties that may be associated with dsRNA uptake. Nevertheless, it would be advisable to test whether there are indeed difficulties in dsRNA uptake in midgut cells before reaching a conclusion that the lack of SID protein hinders RNAi efficiency in *H. halys*.

Another interesting finding was that the dsDNA was not completely degraded by the salivary enzymes (Figures 5 and 6), similar to the observation in *Lygus lineolaris* ^[^ ^47^ ^]^ where separate incubation of a similar amount of dsDNA and dsRNA with saliva did not completely degrade the dsDNA but only the dsRNA. It is possible that HhNSE has a high affinity for degrading dsRNA compared to dsDNA, a pattern reported in *Apolygus lucorum*.^[^ ^59^ ^]^ Also, in the study by Meiss et al., 1999^.[^ ^31^ ^]^ documented that NSEs in *B. mori* showed a predilection for degrading dsRNA over other nucleic acid forms in the insect body. An alternative explanation for the observed results is that the dsDNA may have saturated the available HhNSE, allowing the remaining dsRNA to remain intact and to be protected from HhNSE for some time. This would allow the midgut epithelial cells to take up a sufficient amount of the free dsRNA.

To ensure the durability of dsRNA after oral administration, several studies have successfully used metal ion chelators such as ethylenediaminetetraacetic acid (EDTA),^[^ ^14^ ^]^ chitosan‐based nanoparticles,^[^ ^60^, ^61^ ^]^ and lipid molecules such as Lipofectamine 2000^[^ ^14^ ^]^ to protect dsRNA from degradation by insect nucleases or to increase RNAi efficiency.^[^ ^62^ ^]^ However, using conventional nuclease inhibitors such as EDTA, SDS could also interfere with the health of non‐target organisms as well as the efficiency of key RNAi‐associated nucleases such as Dicer enzymes (acting as dsRNA endoribonucleases) and Argonaute proteins (RNA‐guided endonuclease) upon entering into the epithelial cells of insect gut. Moreover, cytotoxicity to non‐target organisms and later market approval of nanoparticle‐based formulation needs to be concerned.^[^ ^63^, ^64^, ^65^ ^]^ In contrast, dsDNA has been identified as a biologically safe co‐formulant, capable of enhancing the efficiency of RNAi pesticides without, or with minimal, negative environmental impact. Since dsDNA is present in the diet of virtually all living organisms, feeding dsDNA is expected to pose no or low risks, depending on the doses administered.^[^ ^66^ ^]^ Nevertheless, it is important to test the environmental and ecological safety of introducing dsDNA into ecosystems even though it is already widespread in the natural environment. Further research is essential to optimise and develop comprehensive formulations incorporating DNA.

Limitations associated with the use of dsDNA as a co‐formulant could encompass the potential activation of the immune system when dsDNA is administered at high concentrations.^[^ ^67^, ^68^, ^69^ ^]^ Additionally, and of significant concern, is the possibility that dsDNA may compete with dsRNA for uptake and mobility within gut tissues, potentially compromising the efficacy of dsRNA delivery. Therefore, it is crucial to optimize the concentrations of both nucleic acids to fully harness the advantages of this method in enhancing oral RNAi efficiency in *H. halys* and other insect pests representing similar challenges.

Historical applications of DNA, including the use of salmon sperm DNA in environmental RNA extractions and as a blocking agent in hybridization techniques,^[^ ^70^, ^71^ ^]^ have demonstrated the utility of DNA in nucleic acid isolation and hybridization technologies. Our use of DNA to enhance dsRNA stability suggests a potential for improved RNAi‐based pest control strategies.

## 4 Conclusion

In conclusion, our ex vivo analysis revealed significant nuclease activity in the saliva of *H. halys* across various developmental stages tested. Furthermore, RT‐PCR data confirmed high expression of *HhNSEs* in the salivary glands. The addition of dsDNA as a formulant proved effective in reducing dsRNA degradation by these salivary enzymes when incubated ex vivo. These findings were further supported by in vivo assays, which demonstrated that the co‐delivery of dsRNA with dsDNA significantly enhanced gene silencing compared to dsRNA alone. Although no significant increase in mortality rates was observed when dsDNA‐S and dsRNA‐CHC were co‐administered orally, it is hypothesised that there may be a lack of an efficient dsRNA uptake mechanism in *H. halys*. This therefore suggests the need for further research to gain a deeper understanding of the dsRNA uptake mechanism in *H. halys* into gut epithelial cells. These results offer valuable insights into optimizing dsRNA delivery in *H. halys* and highlight potential broader applications for overcoming non‐specific nuclease‐related difficulties to dsRNA delivery in other contexts.

## 5 Experimental Section

### Insect Rearing

*H. halys* was obtained from the laboratory of Katz Biotech AG, Germany, and reared on a diet composed of two to three fresh green beans, few slices of fresh carrots, and husked sunflower seeds, provided ad libitum in a Petri dish. Insects were supplied with water in 15 mL falcon tubes plugged with cotton wicks. This work used the same type of aerated plastic containers (19 × 19 × 19 cm) for all developmental stages which were lined with kitchen paper and maintained in Fitotron HGC1014 Modular Growth Chambers (Weiss Technik GmbH, Reiskirchen, Germany) at 25 °C, 65% humidity, and a photoperiod of 16:8 h. Insects were provided with fresh food three times per week and transferred to new boxes once per week.

### dsRNA and dsDNA Acquisition

For the ex vivo dsRNA degradation studies, beta‐glucuronidase dsRNA (dsRNA‐GUS) of 225 base pairs (bp) was purchased from RNAGreentech LLC (Frisco, USA). dsRNA‐CHC and dsRNA‐GFP used in the in vivo assays was acquired from Genolution AgroRNA (Seoul, Korea), see dsRNA sequences in Table S1, Supporting Information. Two different dsDNAs were used in this study: a 246 bp purified PCR product from *Arabidopsis thaliana, Actin‐2* (dsDNA‐AtACT, accession number NM_001338359.1) and another 102 bp fragment of *H. halys Argonaute‐2 like protein* (dsDNA‐HhAGO2, accession number XM_024358503.1). The degradation experiments were repeated using the shorter *dsRNA‐HhAGO2* fragment, as the slower running speed of dsRNA over dsDNA on the agarose gel resulted in difficulties in the visual separation of the degraded 225 bp dsRNA‐GUS fragment from the 246 bp dsDNA‐AtACT, where the smear of the degraded fragment of dsRNA‐GUS overlapped with the dsDNA‐AtACT (Figure 5A). The primers used are listed in Table S1, Supporting Information. PCR was performed using ALLinTM HiFi DNA Polymerase (highQu GmbH, Kraichtal, Germany), and the amplified dsDNA during the PCR reaction was purified with a Wizard SV Gel and a PCR Clean‐Up System (Promega). Subsequently, the quality of dsDNAs was assessed under UV light by gel electrophoresis at 100 V for 30 min on an in‐gel stained 1% agarose gel with Midori Advance Green (NIPPON Genetics EUROPE, Düren, Germany) in 1x TAE buffer.

### Saliva, Salivary Gland Extract, and Hemolymph Isolation

Saliva collection was restricted to fourth and fifth instar nymphs and adults, as described by^[^ ^72^ ^]^ Peiffer and Felton (2014), due to the practical difficulties of collecting saliva from earlier stages. During sample collection, insects were placed on ice for 5 min, then turned on their backs and fixed on double‐ sided adhesive tape under a stereomicroscope to prevent movement during saliva collection. After 1–2 min at room temperature (≈22–25 °C), bugs released several droplets of saliva at the tip of their proboscis which were collected using nuclease‐free 10 µL pipette tips and pooled in Eppendorf tubes on ice to reach an approximate amount of 0.2 to 1 µL from each adult, 0.2–0.3 µL from each fifth instar, and 0.1–0.2 µL from each fourth instar insect. For each experiment, ≈15–20 insects were used to collect saliva from each developmental stage. Samples were stored on ice until testing.

Salivary glands (including accessory gland, posterior and anterior lobes) from all instars except instar 1 were gently removed from the body by pulling out the head horizontally under ice‐cold PBS (pH 7.4) to minimize cell damage under the stereomicroscope. The glands were separated from the head, rinsed for 5 s in fresh PBS, and transferred to a 1.5 mL tube on ice. Between seven and ten intact salivary glands from 4 to 5 insects per developmental stage were pooled and kept on ice. Next, 3 to 4 µL of PBS were added to the glands and tubes were slightly vortexed for 10 s and centrifuged at 1000 × g for 15 min at 4 °C. The resulting supernatant was collected and used for degradation assays. The procedure for extracting salivary gland extract was partially adapted from Fischer et al 2020.^[^ ^73^ ^]^

Hemolymph was collected from adults and all developmental stages except first and second instar nymphs due to their small body size. An incision was made at the distal portion of the intersegmental membrane between femur and tibia using sterilized spring scissors, and the hemolymph collected with nuclease‐free 10 µL pipette tips was stored in an ice‐cold 1.5 mL tube containing a few crystals (about the size of a sesame seed) of N‐phenylthiourea (Sigma‐Aldrich, Germany) to prevent melanisation.^[^ ^74^ ^]^ ≈20 to 25 insects were utilized in each experiment to obtain hemolymph from various developmental stages tested. Hemocytes were removed by centrifugation at 1000 × g for 8 min at 4 °C,^[^ ^44^ ^]^ and the supernatant was used for the degradation assays. Degradation assays were performed in four replicates for adult saliva and in duplicate for the other sample types (i.e., salivary gland contents and hemolymph). Due to the very limited amount of saliva that can be collected from the fourth and fifth stages, only one measurement was performed.

### Ex Vivo dsRNA Stability Assay

For all ex vivo dsRNA degradation assays, as described above, 2 µL of each of the three freshly collected sample types (saliva, salivary gland extracts, and hemolymph) were incubated undiluted with 20 µL of dsRNA‐GUS (100 ng µL^−1^) at 25 °C using a 1.5 mL Thermomixer (Eppendorf). At ≈1, 10, 30, 60, 120, and 240 min after incubation, aliquots of 5 µL were collected and combined with 2 µL of 1% SDS (sodium dodecyl sulfate) in water to stop the nuclease reaction.^[^ ^34^ ^]^ The samples were immediately subjected to gel electrophoresis analysis after the experiment. Using 1x TAE buffer, an 1% agarose gel was prepared, in‐gel stained with Midori Advance Green (NIPPON Genetics EUROPE, Düren, Germany), and electrophoresed at 100 V for 30 min, followed by visualisation of dsRNA under UV light. In all gels, the lane indicated as dsRNA‐GUS for the positive control contains dsRNA‐GUS from our original stock, not from the experimental incubation with water. A separate incubation of dsRNA‐GUS with water was conducted for each experiment, and the corresponding gel images are presented in Section 4 of the supplementary data.

The intensity of dsRNA bands from agarose gel images was quantified using ImageJ 1.53t software^[^ ^75^ ^]^ and statistically analysed across incubation times using SAS version 9.4 (SAS Institute Inc., Cary, NC, USA), with subsequent data visualization conducted in JMP (version 17.2.0; SAS Institute, Cary, NC). The quantified gel image data (adbi70002-sup-0002-SuppMat.xlsx in Supporting Information) was modelled using PROC MIXED from SAS 9.4, that is, linear mixed modelling and the associated analysis results and graphs are provided in the Supporting Information (sections 4 & 5 in Supporting Information, and statistical analysis report in SI_1.pdf). Although our samples sizes were limited to n = 2, having multiple data points at different times made it possible to model and analyse the data. All the gel images were labelled using the online platform Biorender.com.

### RT‐PCR

Gene expression levels of three different dsRNA degrading nucleases (*HhNSE* (XM_024362815.1)*, HhEri‐1* (XM_024361541.1)*, HhSDN‐1* (XM_014423854.2)) in salivary glands were assessed. To control for inter‐individual variation, 18–20 salivary glands from ten untreated *H. halys* adults were pooled in 400 µL of a DNA/RNA‐protecting solution (Monarch Total RNA Miniprep Kit, New England Biolabs Inc., Germany) for each biological replicate. Total RNA from the homogenized glands was extracted according to the manufacturer's recommendations (Monarch Total RNA Miniprep Kit, New England Biolabs Inc., Frankfurt, Germany). The purified RNA was quantified using a BioPhotometer Plus with a µCuvette G1.0 (Eppendorf). Three biological replicates were evaluated in total.

cDNA synthesis was performed using the qScript cDNA Synthesis kit (VWR International GmbH, Darmstadt, Germany). Primers for RT‐PCR were designed using the PrimeQuest tool (www.idtdna.com), followed by an off‐target prediction check using PrimerBlast (National Center for Biotechnology Information, Bethesda, MD, USA). The primers used in this study are listed in Table S1, Supporting Information. RT‐PCR analysis was conducted with the SYBR Green JumpStart Taq ReadyMix (Sigma‐Aldrich, Germany) using the Bio‐Rad CFX96 PCR detection system. The RT‐PCR experiments were conducted with each biological replicate consisting of two technical replicates to ensure robust and reliable results. After the initial activation step at 94 °C for 2 min, 40 cycles (94 °C for 15 sec, 60 °C for 60 sec, and 72 °C for 30 sec) were performed. RT‐PCR data analysis was conducted through the Pfaffl method,^[^ ^76^ ^]^ which considers primer efficiencies. Average Ct values of *HhEri‐1* were utilized as a calibrator gene for determining the delta Ct values and calculating the ratio of relative gene expression. The raw data and detailed result reports are provided in Supporting Information. For data visualization and statistical analysis, including twofold logarithmic transformation, ANOVA and multiple comparisons with Tukey‐HSD as a post hoc test, JMP (ver. 17.2.0; SAS Institute, Cary, NC) was used.

### dsDNA Competitor Assay

Given that NSE has a broad affinity for nucleic acids,^[^ ^31^ ^]^ we speculated that dsDNA may function as a competitor to protect dsRNA from dsRNases in *H. halys* saliva. To test this hypothesis, we amplified dsDNA originating from *A. thaliana* and *H. halys* (see ‘*dsRNA and dsDNA Acquisition*’ in section 5). At 25 ^°^C, 2 µL of fresh, undiluted saliva was incubated with 20 µL of 100 ng µL^−1^ dsRNA‐GUS and 100 ng µL^−1^ of dsDNA, together diluted in ultrapure, nuclease‐free water (Invitrogen by Thermo Fisher Scientific, Waltham, MA), and aliquots were collected at ≈1, 10, 30, and 60 min. Before incubation, the reaction solution was vortexed and spun for 30 s to ensure proper mixing and to collect the solution at the bottom of the reaction tube after saliva was added. Additionally, 1:0.5, 1:2, and 1:3 ratios of dsRNA‐GUS:dsDNA‐AtACT concentrations were tested. At each time point, 5 µL aliquots were collected and 2 µL of 1% SDS was added to stop the nuclease reaction and left at room temperature. Upon completion of collection of all samples, samples were immediately investigated for dsRNA‐GUS stability by gel electrophoresis without freezing. In this study, the PCR‐amplified dsDNA‐based competitor assays were replicated four times, and the salmon sperm dsDNA‐based assays were replicated twice (see section 4.1.1 in Supporting Information). The sequences of dsRNA involved in this study are provided in Table S2, Supporting Information.

### In Vivo Assays to Assess the Efficiency of the dsRNA Formulation

This study was aimed at evaluating the effect of a dsDNA formulation on oral RNAi efficacy in second instar *H. halys*. Due to the need for large quantities for feeding bioassays, sheared UltraPure Salmon Sperm DNA (dsDNA‐S) from ThermoFisher Scientific, Germany (catalog number: 15 632 011) was utilized as a formulant. The inhibitory effect of dsDNA‐S against *H. halys* salivary nucleases was evaluated in line with the methodology described in ‘*dsDNA Competitor Assay*’ in section 5.

For the bioassays, 200 µL of nucleic acids (i.e., 1:1 ratio of dsDNA‐S and dsRNA‐CHC) were combined with a 1% sucrose solution and red food dye (Dr. Oetker, Germany) and packaged in stretched parafilm sachets. The dye was utilized for two primary objectives: initially, to trace the feeding sites by staining salivary sheaths on the sachet when nymphs attempt to feed on the supplied solution, and second, to observe the stained faeces, both of which confirm successful ingestion of dsRNA. These sachets were immersed in fresh bush bean juice to facilitate recognition by the nymphs, then dried before being offered to the nymphs. The addition of sucrose ensured that the nymphs received the necessary carbohydrates during the incubation period.

We developed a novel staining method to precisely identify the feeding sites of *H. halys* nymphs on parafilm sachets (Figure S1, Supporting Information) as a putative indicator of feeding activity. Typically, before using their stylets to ingest sap, *H. halys* and several other stink bugs secrete a protein‐rich salivary coat that coats the puncture made by their mouthparts on the host.^[^ ^77^ ^]^ We have stained this protein sheath with the aforementioned food dye, and this method makes the coloured spots visible under the microscope and to verify that insects were feeding on the sachets (see Figure S1, Supporting Information).

Three different treatments were tested in the dsRNA feeding assays. Each treatment was replicated six times (n = 6), with each replicate consisting of 5 second instar nymphs. The treatments included: (1) feeding with 20 µg of dsRNA‐GFP as a negative control; (2) 20 µg dsRNA‐CHC targeting *CHC* gene in *H. halys*; and (3) a combination of 20 µg dsRNA‐CHC and 20 µg salmon sperm DNA (dsDNA‐S) (i.e., a 1:1 ratio). Each oral formulation was administered in 200 µL per sachet, fed to groups of 5 second instar nymphs over 72 h in a small Petri dish, see Figure S2, Supporting Information.

In a further experiment, nymphs were either injected with (1) 100 ng dsRNA‐CHC, or (2) 100 ng dsRNA‐GFP as a negative control. Direct dsRNA injections were performed using a Beveled, 35G (NF35BV‐2) needle and an UMP3 ULTRAMICROPUMP microinjector from WPI, USA, all under a stereo microscope. Following the injections, the nymphs were then allowed to feed on fresh bush beans for a duration of 72 h. Each treatment in this experiment was replicated three times (n = 3), with each replicate consisting of RNA from pool of 5 second instar nymphs.

Total RNA was extracted from pooled samples of 5 whole insects per replicate, converted to cDNA, and analysed via RT‐PCR as detailed in ‘*RT‐PCR*’ in section 5, with six replicates per treatment for the feeding assays and three for the injection assays. mRNA levels were normalised to 18S rRNA,^[^ ^29^ ^]^ which served as a reference gene, and analysed using the Pfaffl method.^[^ ^76^ ^]^ Means of gene expression ratio values between treatments were compared using a standard least square model followed by a Tukey‐HSD post‐hoc test using JMP (version 18.2.0; SAS Institute, Cary, NC).

### dsRNA Injection for Survival Analysis

In addition to the analysis of gene expression via RT‐PCR, this work carried out injection assays to assess the mortality caused by dsRNA‐CHC. Second‐instar nymphs were injected as described above. In total, this work carried out three independent experiments with three treatments including the injection of dsRNA‐CHC, dsRNA‐GFP, and nuclease‐free (NF) water. Sample sizes ranged from 14 to 18 individuals per treatment and experimental conditions were the same as described for rearing of the insects. Mortality was assessed over a period of 13 days and cumulative mortality at the end of the trial was corrected for the mortality observed in the NF treatment using Schneider‐Orelli's formula.^[^ ^78^ ^]^ The means of the corrected mortality rates of the different treatments were analysed by ANOVA followed by a Tukey‐HSD post‐hoc test using JMP (version 18.0.2; SAS Institute, Cary, NC).

### dsRNA and dsDNA Feeding Assay for Survival Analysis

To assess the impact of adding dsDNA to the dsRNA‐CHC on the survival of *H. halys* upon oral delivery (i.e., feeding) this work selected second‐stage nymphs and offered 200 µL (100 ng µL^−1^) of dsRNA to a group of five insects each following the procedure described for the RT‐PCR experiments. Following a 72‐h feeding period, the surviving insects were transferred to a new plastic jar (dimensions of 7.5 cm in height and 8.5 cm in diameter) with a mesh‐covered lid and supplied them with fresh green bush beans. In this study, seven independent experiments were conducted, each comprising a specific set of treatments. experiments 1, 2, 3, 5, and 6 comprised all seven treatments, namely dsRNA‐CHC, dsRNA‐CHC plus dsDNA‐S (1:1), dsRNA‐GFP, dsRNA‐GFP plus dsDNA‐S (1:1), dsDNA‐S, 1% sucrose, and an untreated control. Experiment 4 comprised five treatments, with the exclusion of dsRNA‐CHC plus dsDNA‐S and dsRNA‐GFP. Similarly, experiment 7 comprised five treatments, with the exclusion of dsRNA‐CHC plus dsDNA‐S, and included only dsDNA‐S, 1% sucrose, and the untreated control. To confirm that the insects were feeding on the solution provided, this work checked for the presence of stained feeding marks on the parafilm sachet (note that in this experiment the food dye was directly added into the feeding solution, as mentioned in ‘*In Vivo Assays to Assess the Efficiency of the dsRNA Formulation*’ in section 5). Survival data were then recorded over a 14‐day period, see adbi70002-sup-0003-SuppMat.xlsx in Supporting Information. The mortality data were ultimately subjected to visualisation and analysis using a GLM in JMP (version 17.2.0; SAS Institute, Cary, NC).

## Conflict of Interest

The authors declare no conflict of interest.

## Author Contributions

Conceptualization: GP, AK and VPSA: Data Curation: VPSA: Formal Analysis: VPSA: Funding Acquisition: AK, and GP: Investigation: VPSA: Methodology: AK, GP, and VPSA: Project administration: AK and VPSA: Resources: AK and GP: Supervision: AK, GP, and VPSA: Validation: AK, GP, and VPSA: Writing ‐ Original Draft Preparation: VPSA: Writing ‐ Review & Editing: AK, GP, and VPSA: Visualisation: VPSA:

## Supporting information



Supporting Information

Supporting Information

Supporting Information

Supporting Information

Supporting Information

Supporting Information

Supporting Information

Supporting Information

Supporting Information

## Data Availability

The data that support the findings of this study are available in the supplementary material of this article.
