# Supplementary material for: Double‐Stranded DNA Reduces dsRNA Degradation in the Saliva and Significantly Enhanced RNAi‐Mediated Gene Silencing in Halyomorpha halys
Source: Adv Biol (Weinh). 2025 Aug 17;9(9):e00698. doi: 10.1002/adbi.202400698 (PMC12447125; doi:10.1002/adbi.202400698)
Supplement: Supplementary file 4 — Supporting Information [file ADBI-9-e00698-s006.pdf]

ADVANCED BIOLOGY

Supporting Information

for *Adv. Biology*, DOI 10.1002/adbi.202400698

Double-Stranded DNA Reduces dsRNA Degradation in the Saliva and Significantly Enhanced RNAi-Mediated Gene Silencing in *Halyomorpha halys*

*Venkata Partha Sarathi Amineni, Georg Petschenka and Aline Koch**

The SAS System

The Mixed Procedure

Model Information	
Data Set	WORK.GEL_DATA
Dependent Variable	Volume
Covariance Structure	Variance Components
Estimation Method	REML
Residual Variance Method	Profile
Fixed Effects SE Method	Kenward-Roger
Degrees of Freedom Method	Kenward-Roger

Class Level Information

Class	Levels	Values
Treatment	2	dsRNA + Hemolymph dsRNA+water
Time	5	0 1 10 30 60
Gels	4	6 9 10 11
Life_stage	5	2 3 4 5 Adult
Repetition	1	A

Dimensions	
Covariance Parameters	3
Columns in X	23
Columns in Z	8
Subjects	1
Max Obs per Subject	78

Number of Observations	
Number of Observations Read	90
Number of Observations Used	78
Number of Observations Not Used	12

Iteration History

Iteration	Evaluations	-2 Res Log Like	Criterion
0	1	1277.62911482	
1	2	1249.67564415	0.00169842
2	1	1249.46555118	0.00017579
3	1	1249.42060929	0.00000813
4	1	1249.41748012	0.00000005
5	1	1249.41745803	0.00000000

Convergence criteria met but final Hessian is not positive definite.

Covariance Parameter Estimates	
**Cov Parm**	**Estimate**
Gels	13551123
Gels*Repetition	4940367
Residual	9734101

Fit Statistics	
-2 Res Log Likelihood	1249.4
AIC (Smaller is Better)	1255.4
AICC (Smaller is Better)	1255.8
BIC (Smaller is Better)	1253.6

Solution for Fixed Effects

Effect	Treatment	Life_stage	Time	Estimate	Standard Error	DF	t Value	Pr > |t|
Intercept				19940	2898.90	6.79	6.88	0.0003
Treatment	dsRNA + Hemolymph			-3709.75	1811.65	61.5	-2.05	0.0449
Treatment	dsRNA+water			0	.	.	.	.
Life_stage		2		-8376.43	2761.79	56.7	-3.03	0.0036
Life_stage		3		-8376.43	2761.79	56.7	-3.03	0.0036
Life_stage		4		-6371.50	2628.67	61.7	-2.42	0.0183
Life_stage		5		-787.70	1801.31	60.9	-0.44	0.6634
Life_stage		Adult		0	.	.	.	.
Treatment*Life_stage	dsRNA + Hemolymph	2		5202.65	2300.13	61.2	2.26	0.0273
Treatment*Life_stage	dsRNA + Hemolymph	3		1301.46	2300.13	61.2	0.57	0.5736
Treatment*Life_stage	dsRNA + Hemolymph	4		-3204.04	2502.93	62.5	-1.28	0.2052
Treatment*Life_stage	dsRNA + Hemolymph	5		-3343.09	2382.90	60.9	-1.40	0.1657
Treatment*Life_stage	dsRNA + Hemolymph	Adult		0	.	.	.	.
Treatment*Life_stage	dsRNA+water	2		0	.	.	.	.
Treatment*Life_stage	dsRNA+water	3		0	.	.	.	.
Treatment*Life_stage	dsRNA+water	4		0	.	.	.	.
Treatment*Life_stage	dsRNA+water	5		0	.	.	.	.
Treatment*Life_stage	dsRNA+water	Adult		0	.	.	.	.
Time			0	-3225.22	1370.46	61.2	-2.35	0.0218
Time			1	-978.42	1070.13	60.9	-0.91	0.3642
Time			10	-308.03	1070.13	60.9	-0.29	0.7744
Time			30	285.05	1070.13	60.9	0.27	0.7909
Time			60	0	.	.	.	.

Type 3 Tests of Fixed Effects				
**Effect**	**Num DF**	**Den DF**	**F Value**	**Pr > F**
**Treatment**	1	61.7	19.24	<.0001
**Life_stage**	4	61	5.54	0.0007
**Treatment*Life_stage**	4	61.5	4.78	0.0020
**Time**	4	61	1.91	0.1198

Least Squares Means						
**Effect**	**Treatment**	**Estimate**	**Standard Error**	**DF**	**t Value**	**Pr > |t|**
**Treatment**	dsRNA + Hemolymph	10594	2222.28	2.79	4.77	0.0206
**Treatment**	dsRNA+water	14312	2223.83	2.8	6.44	0.0094

Differences of Least Squares Means							
**Effect**	**Treatment**	**Treatment**	**Estimate**	**Standard Error**	**DF**	**t Value**	**Pr > |t|**
**Treatment**	dsRNA + Hemolymph	dsRNA+water	-3718.35	847.74	61.7	-4.39	<.0001

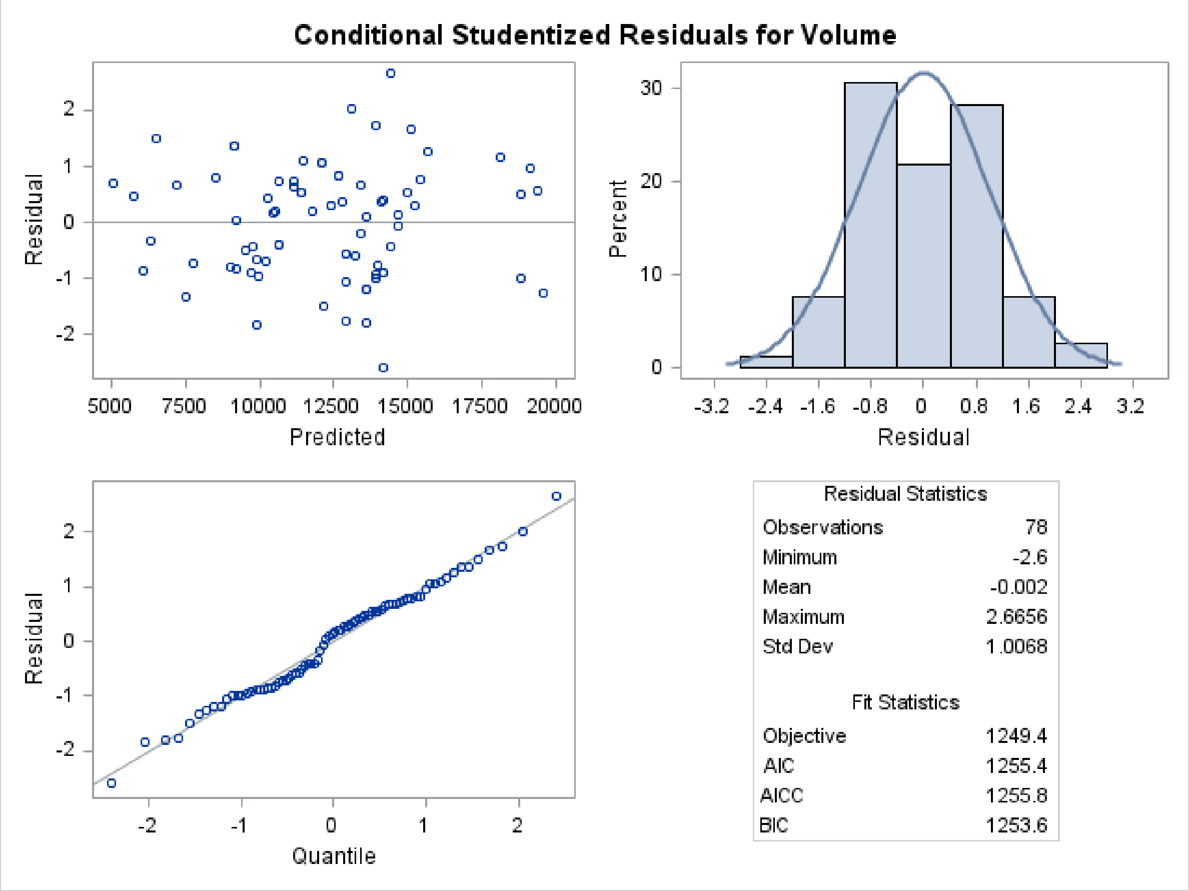

The SAS System

The Mixed Procedure

Model Information	
Data Set	WORK.GEL_DATA
Dependent Variable	Volume
Covariance Structure	Variance Components
Estimation Method	REML
Residual Variance Method	Profile
Fixed Effects SE Method	Kenward-Roger
Degrees of Freedom Method	Kenward-Roger

Class Level Information		
Class	Levels	Values
Treatment	3	dsRNA + DNA dsRNA+saliva dsRNA+water
Time	5	0 1 10 30 60
Gels	3	1 2 3
Life_stage	1	Adult
Repetition	1	A

Dimensions	
Covariance Parameters	2
Columns in X	9
Columns in Z	3
Subjects	1
Max Obs per Subject	34

Number of Observations	
Number of Observations Read	39
Number of Observations Used	34
Number of Observations Not Used	5

Iteration History			
Iteration	Evaluations	-2 Res Log Like	Criterion
0	1	522.86979103	
1	2	506.06037367	0.00002689
2	1	506.05370833	0.00000056
3	1	506.05357818	0.00000000

Convergence criteria met.

Covariance Parameter Estimates	

Cov Parm	Estimate
Gels	7460110
Residual	4058731

Fit Statistics	
-2 Res Log Likelihood	506.1
AIC (Smaller is Better)	510.1
AICC (Smaller is Better)	510.6
BIC (Smaller is Better)	508.3

Solution for Fixed Effects

Effect	Treatment	Time	Estimate	Standard Error	DF	t Value	Pr > |t|
Intercept			8435.62	1859.35	3.4	4.54	0.0152
Treatment	dsRNA + DNA		-3139.04	963.43	25.2	-3.26	0.0032
Treatment	dsRNA+saliva		-6878.57	963.43	25.2	-7.14	<.0001
Treatment	dsRNA+water		0	.	.	.	.
Time		0	2497.40	1776.12	25.2	1.41	0.1719
Time		1	1950.69	1007.31	25	1.94	0.0642
Time		10	1276.76	1007.31	25	1.27	0.2167
Time		30	1255.29	1007.31	25	1.25	0.2243
Time		60	0	.	.	.	.

Type 3 Tests of Fixed Effects

Effect	Num DF	Den DF	F Value	Pr > F
Treatment	2	25.1	26.86	<.0001
Time	4	25	1.15	0.3562

Least Squares Means

Effect	Treatment	Estimate	Standard Error	DF	t Value	Pr > |t|
Treatment	dsRNA + DNA	6692.60	1713.45	2.49	3.91	0.0414
Treatment	dsRNA+saliva	2953.07	1713.45	2.49	1.72	0.2017
Treatment	dsRNA+water	9831.64	1712.91	2.48	5.74	0.0174

Differences of Least Squares Means

Effect	Treatment	Treatment	Estimate	Standard Error	DF	t Value	Pr > |t|	Adjustment	Adj P
Treatment	dsRNA + DNA	dsRNA+saliva	3739.53	822.47	25	4.55	0.0001	Tukey-Kramer	0.0003
Treatment	dsRNA + DNA	dsRNA+water	-3139.04	963.43	25.2	-3.26	0.0032	Tukey-Kramer	0.0087
Treatment	dsRNA+saliva	dsRNA+water	-6878.57	963.43	25.2	-7.14	<.0001	Tukey-Kramer	<.0001

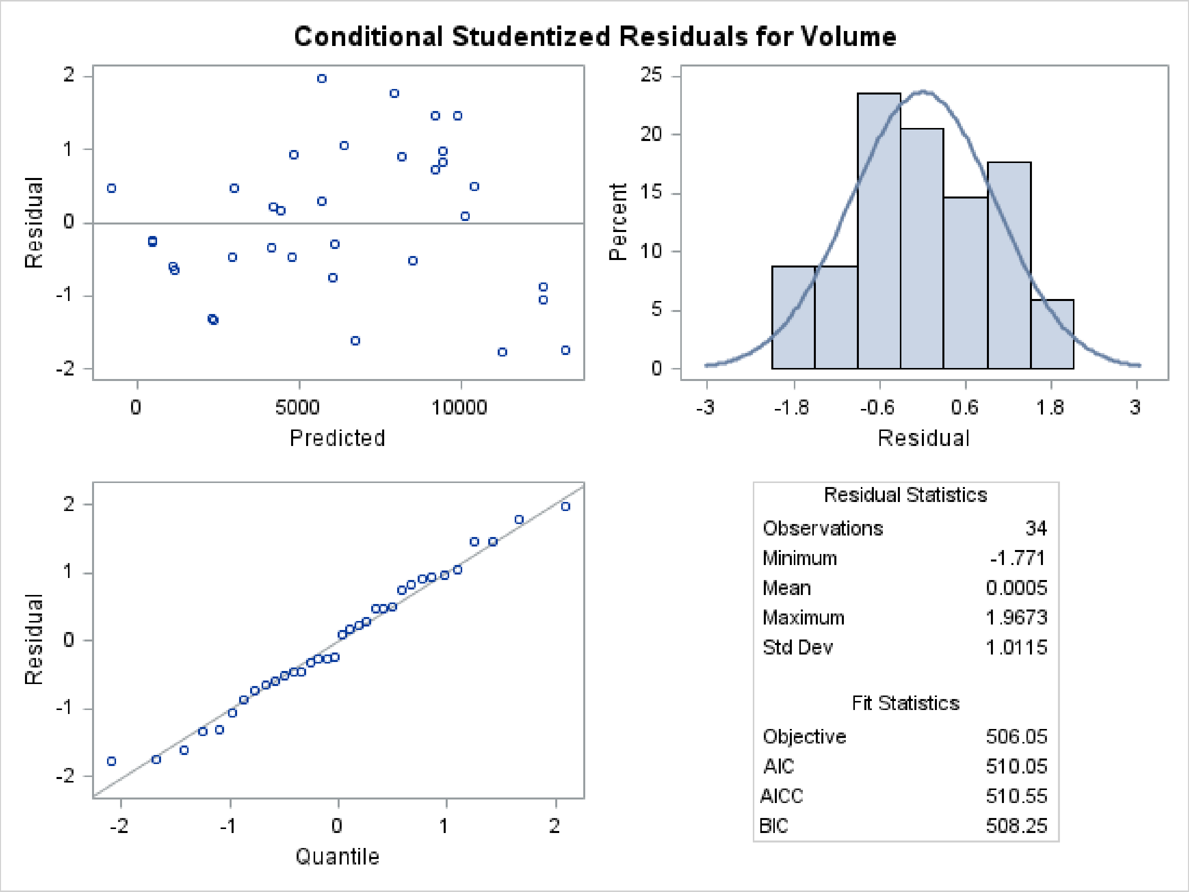
Conditional Studentized Residuals for Volume
Residual
Predicted
0
5000
10000
Percent
Residual
-3
-1.8
-0.6
0.6
1.8
3
Quantile
Residual Statistics
Observations 34
Minimum -1.771
Mean 0.0005
Maximum 1.9673
Std Dev 1.0115
Fit Statistics
Objective 506.05
AIC 510.05
AICC 510.55
BIC 508.25

The SAS System

The Mixed Procedure

Model Information	
Data Set	WORK.GEL_DATA
Dependent Variable	Volume
Covariance Structure	Variance Components
Estimation Method	REML
Residual Variance Method	Profile
Fixed Effects SE Method	Kenward-Roger
Degrees of Freedom Method	Kenward-Roger

Class Level Information		
Class	Levels	Values
Treatment	3	dsRNA + DNA dsRNA+saliva dsRNA+water
Time	5	0 1 10 30 60
Gels	3	1 2 3
Life_stage	1	Adult
Repetition	1	A

Dimensions	
Covariance Parameters	2
Columns in X	9
Columns in Z	3
Subjects	1
Max Obs per Subject	34

Number of Observations	
Number of Observations Read	39
Number of Observations Used	34
Number of Observations Not Used	5

Iteration History			
Iteration	Evaluations	-2 Res Log Like	Criterion
0	1	522.86979103	
1	2	506.06037367	0.00002689
2	1	506.05370833	0.00000056
3	1	506.05357818	0.00000000

Convergence criteria met.

Covariance Parameter Estimates	

Cov Parm	Estimate
Gels	7460110
Residual	4058731

Fit Statistics	
-2 Res Log Likelihood	506.1
AIC (Smaller is Better)	510.1
AICC (Smaller is Better)	510.6
BIC (Smaller is Better)	508.3

Solution for Fixed Effects

Effect	Treatment	Time	Estimate	Standard Error	DF	t Value	Pr > |t|
Intercept			8435.62	1859.35	3.4	4.54	0.0152
Treatment	dsRNA + DNA		-3139.04	963.43	25.2	-3.26	0.0032
Treatment	dsRNA+saliva		-6878.57	963.43	25.2	-7.14	<.0001
Treatment	dsRNA+water		0	.	.	.	.
Time		0	2497.40	1776.12	25.2	1.41	0.1719
Time		1	1950.69	1007.31	25	1.94	0.0642
Time		10	1276.76	1007.31	25	1.27	0.2167
Time		30	1255.29	1007.31	25	1.25	0.2243
Time		60	0	.	.	.	.

Type 3 Tests of Fixed Effects

Effect	Num DF	Den DF	F Value	Pr > F
Treatment	2	25.1	26.86	<.0001
Time	4	25	1.15	0.3562

Least Squares Means

Effect	Treatment	Estimate	Standard Error	DF	t Value	Pr > |t|
Treatment	dsRNA + DNA	6692.60	1713.45	2.49	3.91	0.0414
Treatment	dsRNA+saliva	2953.07	1713.45	2.49	1.72	0.2017
Treatment	dsRNA+water	9831.64	1712.91	2.48	5.74	0.0174

Differences of Least Squares Means

Effect	Treatment	Treatment	Estimate	Standard Error	DF	t Value	Pr > |t|	Adjustment	Adj P
Treatment	dsRNA + DNA	dsRNA+saliva	3739.53	822.47	25	4.55	0.0001	Tukey-Kramer	0.0003
Treatment	dsRNA + DNA	dsRNA+water	-3139.04	963.43	25.2	-3.26	0.0032	Tukey-Kramer	0.0087
Treatment	dsRNA+saliva	dsRNA+water	-6878.57	963.43	25.2	-7.14	<.0001	Tukey-Kramer	<.0001

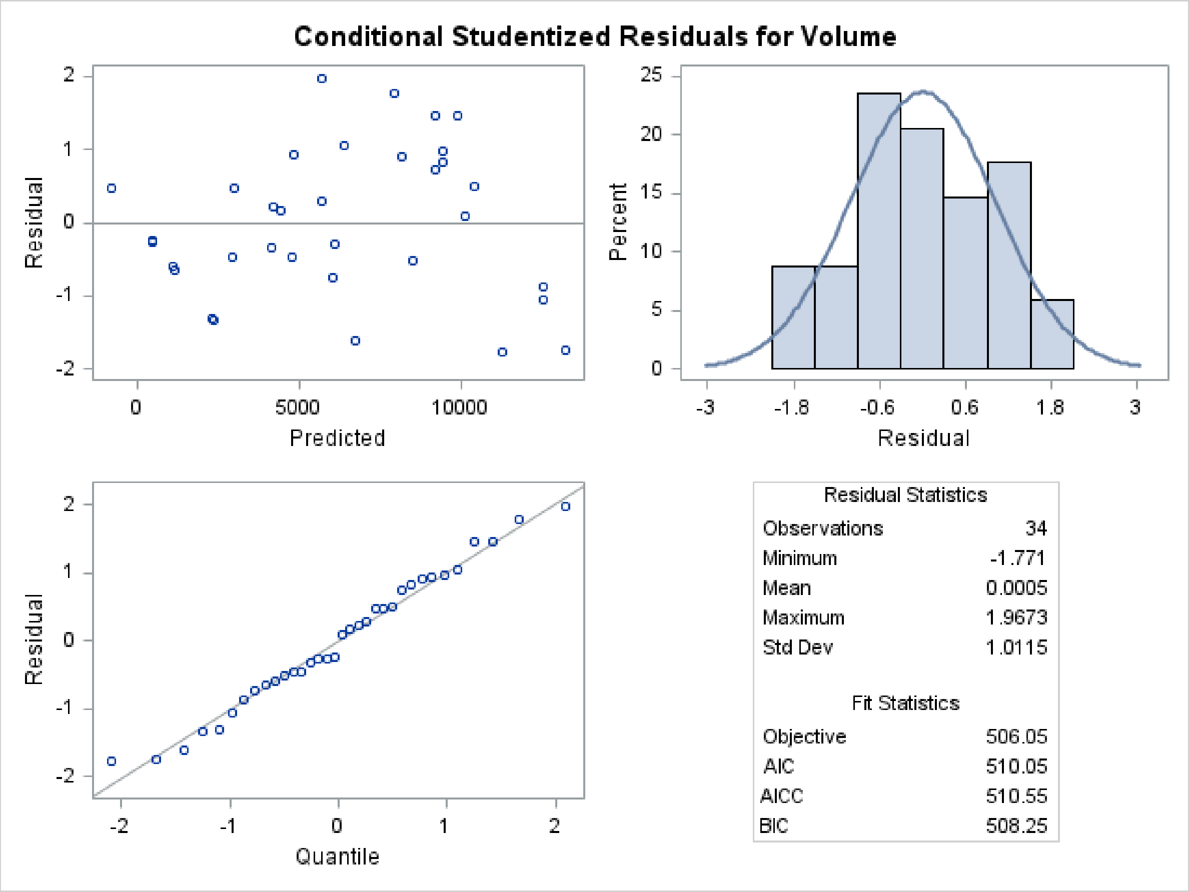
Conditional Studentized Residuals for Volume
Residual
Predicted
Percent
Residual
Residual
Quantile
Residual Statistics
Observations 34
Minimum -1.771
Mean 0.0005
Maximum 1.9673
Std Dev 1.0115
Fit Statistics
Objective 506.05
AIC 510.05
AICC 510.55
BIC 508.25

The SAS System

The Mixed Procedure

Model Information	
Data Set	WORK.GEL_DATA
Dependent Variable	Volume
Covariance Structure	Variance Components
Estimation Method	REML
Residual Variance Method	Profile
Fixed Effects SE Method	Kenward-Roger
Degrees of Freedom Method	Kenward-Roger

Class Level Information		
Class	Levels	Values
Treatment	2	dsRNA + SG dsRNA+water
Time	5	0 1 10 30 60
Gels	5	4 5 6 7 8
Life_stage	5	2 3 4 5 Adult
Repetition	1	A

Dimensions	
Covariance Parameters	2
Columns in X	8
Columns in Z	5
Subjects	1
Max Obs per Subject	88

Number of Observations	
Number of Observations Read	90
Number of Observations Used	88
Number of Observations Not Used	2

Iteration History			
Iteration	Evaluations	-2 Res Log Like	Criterion
0	1	1563.75983248	
1	3	1533.00315077	0.00003938
2	1	1532.97271701	0.00000169
3	1	1532.97151484	0.00000000

Convergence criteria met.

Covariance Parameter Estimates	

Cov Parm	Estimate
Gels	4616109
Residual	5537784

Fit Statistics	
-2 Res Log Likelihood	1533.0
AIC (Smaller is Better)	1537.0
AICC (Smaller is Better)	1537.1
BIC (Smaller is Better)	1536.2

Solution for Fixed Effects

Effect	Treatment	Time	Estimate	Standard Error	DF	t Value	Pr > |t|
Intercept			10922	1141.51	6.54	9.57	<.0001
Treatment	dsRNA + SG		-11818	530.11	78	-22.29	<.0001
Treatment	dsRNA+water		0	.	.	.	.
Time		0	132.44	981.55	78	0.13	0.8930
Time		1	546.51	755.19	78.1	0.72	0.4714
Time		10	583.59	744.16	78	0.78	0.4353
Time		30	159.96	744.16	78	0.21	0.8304
Time		60	0	.	.	.	.

Type 3 Tests of Fixed Effects

Effect	Num DF	Den DF	F Value	Pr > F
Treatment	1	78	496.95	<.0001
Time	4	78	0.23	0.9189

Least Squares Means

Effect	Treatment	Estimate	Standard Error	DF	t Value	Pr > |t|
Treatment	dsRNA + SG	-611.22	1058.49	4.86	-0.58	0.5894
Treatment	dsRNA+water	11206	1034.96	4.44	10.83	0.0002

Differences of Least Squares Means

Effect	Treatment	Treatment	Estimate	Standard Error	DF	t Value	Pr > |t|	Adjustment	Adj P
Treatment	dsRNA + SG	dsRNA+water	-11818	530.11	78	-22.29	<.0001	Tukey-Kramer	<.0001

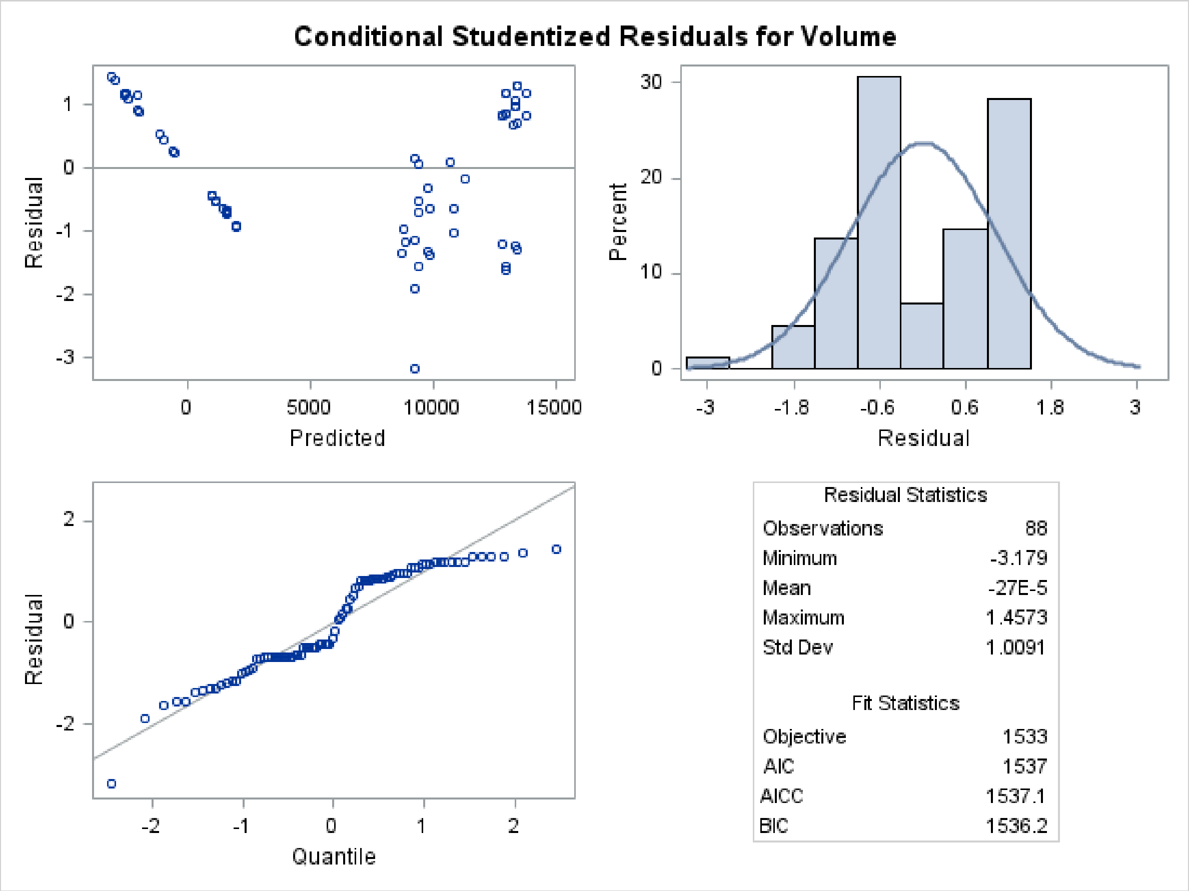
Conditional Studentized Residuals for Volume
Residual
Predicted
0
5000
10000
15000
Percent
Residual
-3
-1.8
-0.6
0.6
1.8
3
Quantile
Residual Statistics
Observations 88
Minimum -3.179
Mean -27E-5
Maximum 1.4573
Std Dev 1.0091
Fit Statistics
Objective 1533
AIC 1537
AICC 1537.1
BIC 1536.2
