# Supplementary material for: Double‐Stranded DNA Reduces dsRNA Degradation in the Saliva and Significantly Enhanced RNAi‐Mediated Gene Silencing in Halyomorpha halys
Source: Adv Biol (Weinh). 2025 Aug 17;9(9):e00698. doi: 10.1002/adbi.202400698 (PMC12447125; doi:10.1002/adbi.202400698)
Supplement: Supplementary file 5 — Supporting Information [file ADBI-9-e00698-s004.pdf]

ADVANCED
BIOLOGY

Supporting Information

for *Adv. Biology*, DOI 10.1002/adbi.202400698

Double-Stranded DNA Reduces dsRNA Degradation in the Saliva and Significantly Enhanced RNAi-Mediated Gene Silencing in *Halyomorpha halys*

*Venkata Partha Sarathi Amineni, Georg Petschenka and Aline Koch**

Response Log2[Gene expression ratio] 2

Whole Model

Actual by Predicted Plot

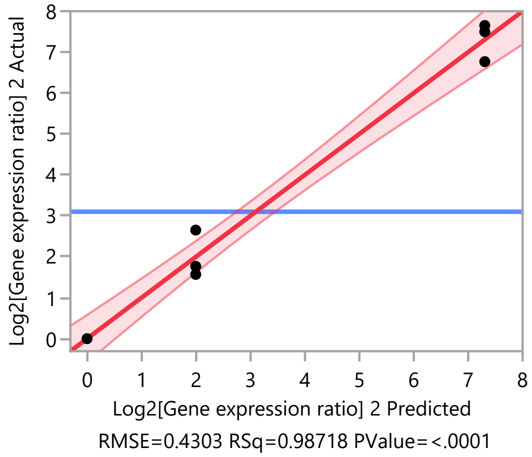

Residual by Predicted Plot

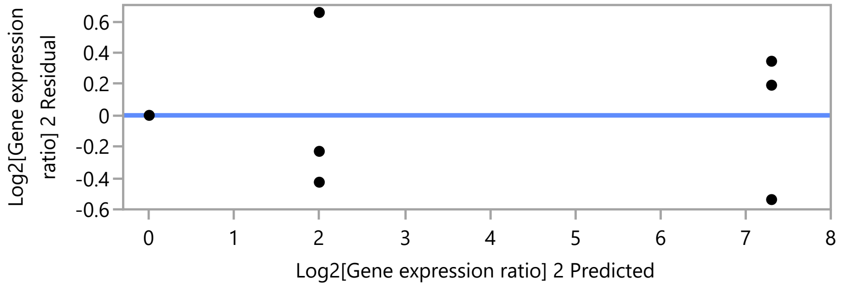

Summary of Fit

RSquare	0.987179
RSquare Adj	0.982905
Root Mean Square Error	0.4303
Mean of Response	3.100497
Observations (or Sum Wgts)	9

Analysis of Variance

Source	DF	Sum of Squares	Mean Square	F Ratio
Model	2	85.537039	42.7685	230.9836
Error	6	1.110950	0.1852	**Prob > F**
C. Total	8	86.647989		<.0001*

Effect Tests

Source	Nparm	DF	Sum of Squares	F Ratio	Prob > F
Treatment	2	2	85.537039	230.9836	<.0001*

Response Log2[Gene expression ratio] 2

Treatment

Leverage Plot

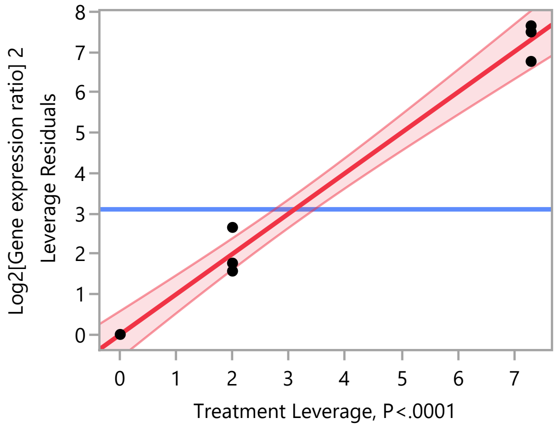

Least Squares Means Table

Level	Least Sq Mean	Std Error	Mean
HhEri 1	1.3323e-15	0.24843395	1.33e-15
HhSDN	1.9962464	0.24843395	1.99625
HhNSE	7.3052438	0.24843395	7.30524

Multiple Comparisons for Treatment

Least Squares Means Estimates

Treatment	Estimate	Std Error	DF	Lower 95%	Upper 95%	Arithmetic Mean Estimate	N
HhEri 1	1.3323e-15	0.24843395	6	-0.607896	0.6078960	0.0000000	3
HhSDN	1.9962464	0.24843395	6	1.388350	2.6041423	1.9962464	3
HhNSE	7.3052438	0.24843395	6	6.697348	7.9131397	7.3052438	3

Least Squares Means Plot

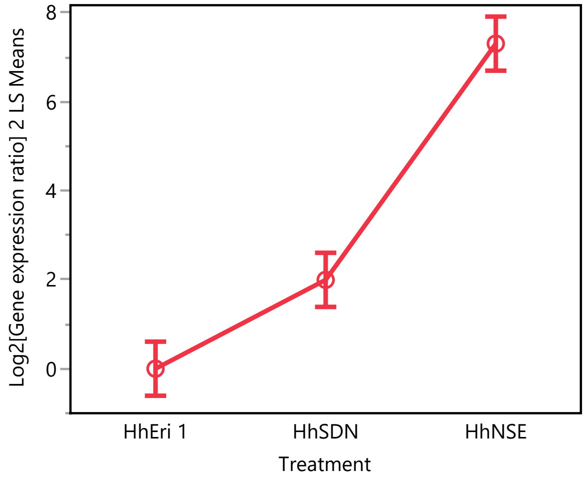

Tukey HSD All Pairwise Comparisons

Quantile = 3.06815, Adjusted DF = 6.0, Adjustment = Tukey

Response Log2[Gene expression ratio] 2

Multiple Comparisons for Treatment

Tukey HSD All Pairwise Comparisons

All Pairwise Differences

Treatment	-Treatment	Difference	Std Error	t Ratio	Prob>|t|	Lower 95%	Upper 95%
HhEri 1	HhSDN	-1.99625	0.3513387	-5.68	0.0031*	-3.07421	-0.91829
HhEri 1	HhNSE	-7.30524	0.3513387	-20.79	<.0001*	-8.38320	-6.22728
HhSDN	HhNSE	-5.30900	0.3513387	-15.11	<.0001*	-6.38696	-4.23104

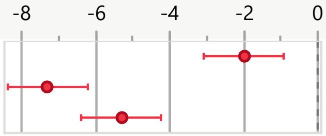

All Pairwise Comparisons Scatterplot

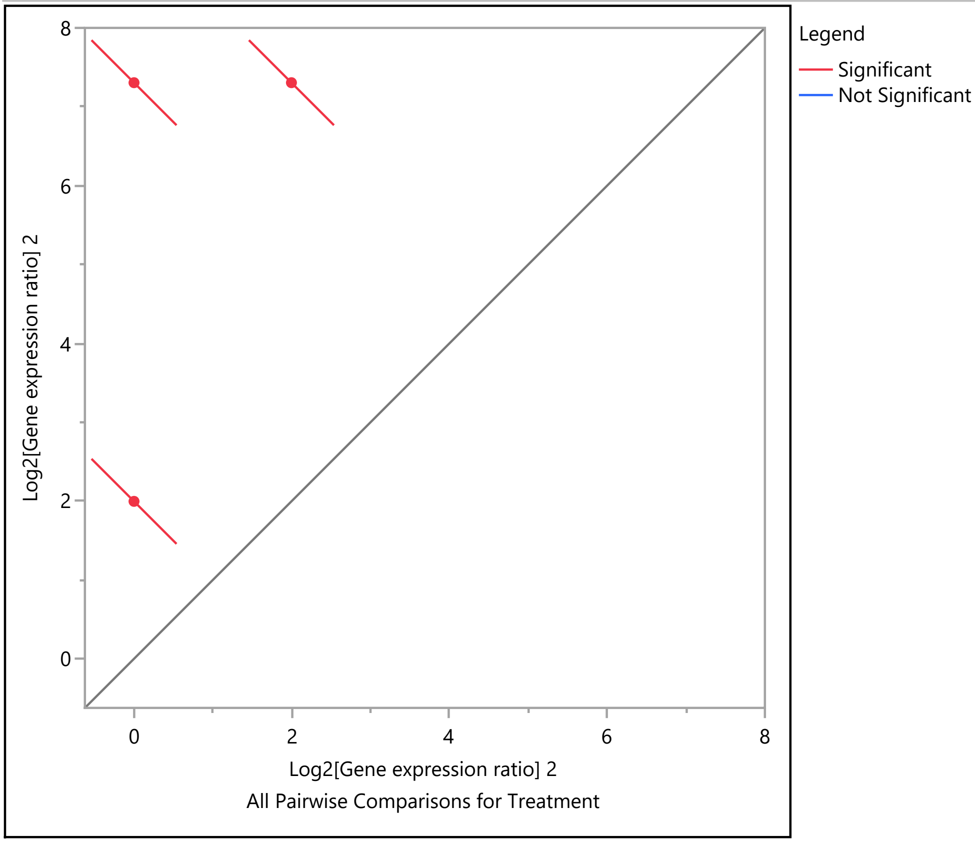

All Pairwise Comparisons for Treatment
