# Supplementary material for: Double‐Stranded DNA Reduces dsRNA Degradation in the Saliva and Significantly Enhanced RNAi‐Mediated Gene Silencing in Halyomorpha halys
Source: Adv Biol (Weinh). 2025 Aug 17;9(9):e00698. doi: 10.1002/adbi.202400698 (PMC12447125; doi:10.1002/adbi.202400698)

ADVANCED
BIOLOGY

Supporting Information

for *Adv. Biology*, DOI 10.1002/adbi.202400698

Double-Stranded DNA Reduces dsRNA Degradation in the Saliva and Significantly Enhanced RNAi-Mediated Gene Silencing in *Halyomorpha halys*

*Venkata Partha Sarathi Amineni, Georg Petschenka and Aline Koch**

Response Gene expression ratio

Whole Model

Actual by Predicted Plot

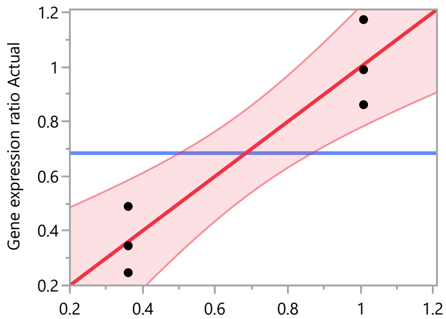

Summary of Fit

RSquare	0.888798
RSquare Adj	0.860997
Root Mean Square Error	0.140261
Mean of Response	0.68424
Observations (or Sum Wgts)	6

Analysis of Variance

Source	DF	Sum of Squares	Mean Square	F Ratio
Model	1	0.62896263	0.628963	31.9705
Error	4	0.07869282	0.019673	Prob > F
C. Total	5	0.70765545		0.0048*

Parameter Estimates

Term	Estimate	Std Error	t Ratio	Prob>|t|
Intercept	0.68424	0.057261	11.95	0.0003*
Treatment[dsRNA-GFP]	0.3237701	0.057261	5.65	0.0048*

Residual by Predicted Plot

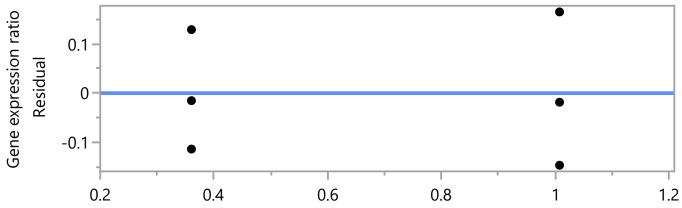

Treatment

Leverage Plot

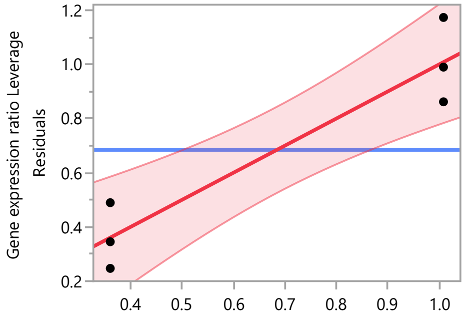

Least Squares Means Table

Level	Least Sq Mean	Std Error	0.2 0.4 0.6 0.8 1.0 1.2	Mean
dsRNA-GFP	1.00801	0.0810	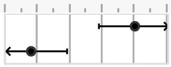	1.0080
dsRNA-CHC Inj.	0.36047	0.0810		0.3605

Response Gene expression ratio

Multiple Comparisons for Treatment

Least Squares Means Estimates

Treatment	Estimate	Std Error	DF	Lower 95%	Upper 95%	Arithmetic Mean Estimate	N
dsRNA-GFP	1.0080102	0.08097984	4	0.78317408	1.2328463	1.0080102	3
dsRNA-CHC Inj.	0.3604699	0.08097984	4	0.13563380	0.5853060	0.3604699	3

Tukey HSD All Pairwise Comparisons

Quantile = 2.77645, Adjusted DF = 4.0, Adjustment = Tukey

All Pairwise Differences

Treatment	-Treatment	Difference	Std Error	t Ratio	Prob>|t|	Lower 95%	Upper 95%
dsRNA-GFP	dsRNA-CHC Inj.	0.6475403	0.1145228	5.65	0.0048*	0.3295739	0.9655067

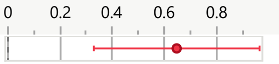

All Pairwise Comparisons Scatterplot

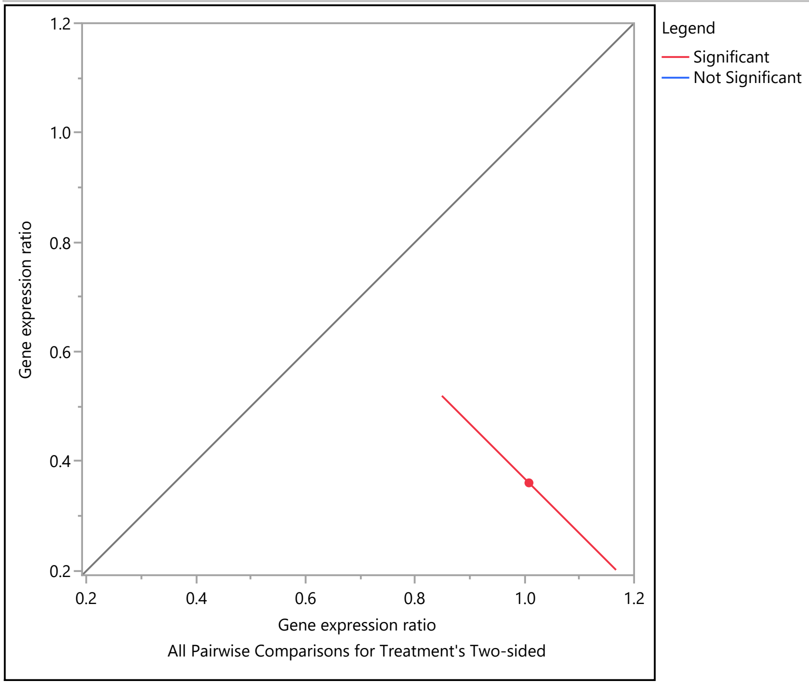

Supplement: Supplementary file 6 — Supporting Information [file ADBI-9-e00698-s007.pdf]
