# Supplementary material for: Double‐Stranded DNA Reduces dsRNA Degradation in the Saliva and Significantly Enhanced RNAi‐Mediated Gene Silencing in Halyomorpha halys
Source: Adv Biol (Weinh). 2025 Aug 17;9(9):e00698. doi: 10.1002/adbi.202400698 (PMC12447125; doi:10.1002/adbi.202400698)

ADVANCED BIOLOGY

Supporting Information

for *Adv. Biology*, DOI 10.1002/adbi.202400698

Double-Stranded DNA Reduces dsRNA Degradation in the Saliva and Significantly Enhanced RNAi-Mediated Gene Silencing in *Halyomorpha halys*

*Venkata Partha Sarathi Amineni, Georg Petschenka and Aline Koch**

Response Gene expression ratio

Whole Model

Actual by Predicted Plot

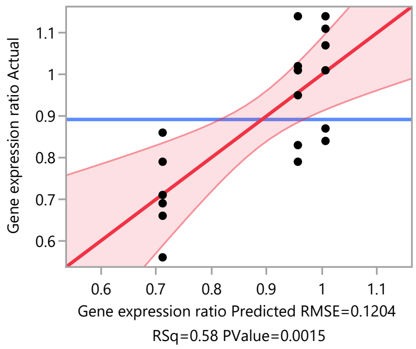

Summary of Fit

RSquare	0.578922
RSquare Adj	0.522778
Root Mean Square Error	0.12043
Mean of Response	0.891667
Observations (or Sum Wgts)	18

Analysis of Variance

Source	DF	Sum of Squares	Mean Square	F Ratio
Model	2	0.29910000	0.149550	10.3114
Error	15	0.21755000	0.014503	**Prob > F**
C. Total	17	0.51665000		0.0015*

Effect Tests

Source	Nparm	DF	Sum of Squares	F Ratio	Prob > F
Treatment	2	2	0.29910000	10.3114	0.0015*

Residual by Predicted Plot

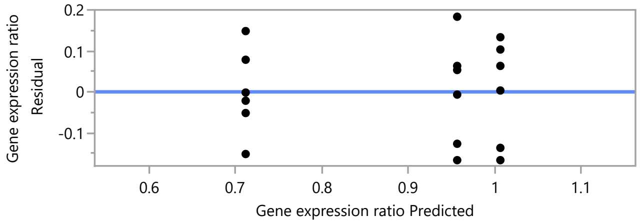

Treatment

Leverage Plot

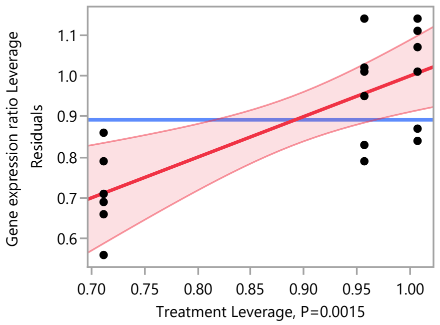

Least Squares Means Table

Level	Least Sq Mean	Std Error	Mean
dsRNA-GFP	1.00667	0.0492	1.0067
dsRNA-CHC	0.95667	0.0492	0.9567
dsRNA-CHC + DNA	0.71167	0.0492	0.7117

Multiple Comparisons for Treatment

Least Squares Means Estimates

Treatment	Estimate	Std Error	DF	Lower 95%	Upper 95%	Arithmetic Mean Estimate	N
dsRNA-GFP	1.0066667	0.04916525	15	0.90187341	1.1114599	1.0066667	6
dsRNA-CHC	0.9566667	0.04916525	15	0.85187341	1.0614599	0.9566667	6
dsRNA-CHC + DNA	0.7116667	0.04916525	15	0.60687341	0.8164599	0.7116667	6

Tukey HSD All Pairwise Comparisons

Quantile = 2.59747, Adjusted DF = 15.0, Adjustment = Tukey

All Pairwise Differences

Treatment	-Treatment	Difference	Std Error	t Ratio	Prob>|t|	Lower 95%	Upper 95%
dsRNA-GFP	dsRNA-CHC	0.0500000	0.0695302	0.72	0.7562	-0.130603	0.2306026
dsRNA-GFP	dsRNA-CHC + DNA	0.2950000	0.0695302	4.24	0.0019*	0.114397	0.4756026
dsRNA-CHC	dsRNA-CHC + DNA	0.2450000	0.0695302	3.52	0.0081*	0.064397	0.4256026

Response Gene expression ratio

Multiple Comparisons for Treatment

Tukey HSD All Pairwise Comparisons

All Pairwise Comparisons Scatterplot

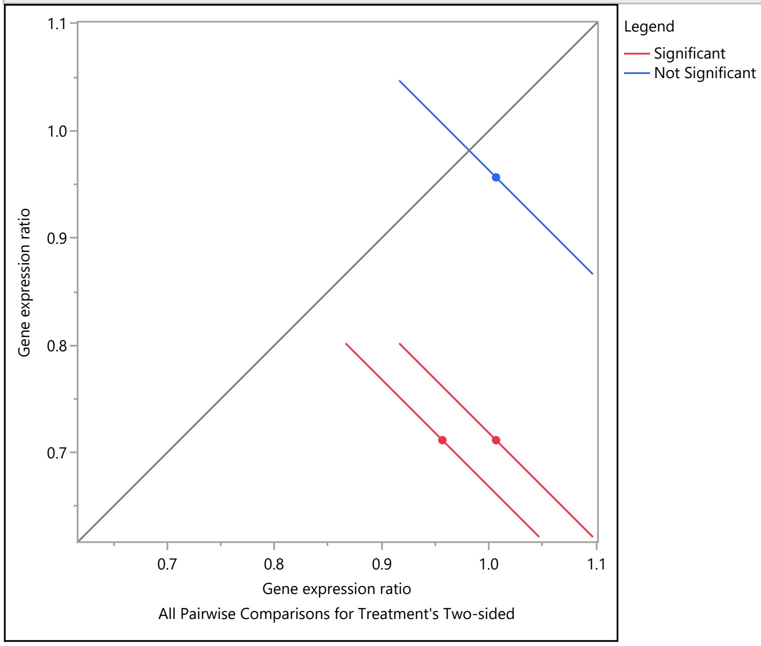

Supplement: Supplementary file 7 — Supporting Information [file ADBI-9-e00698-s008.pdf]
