# Supplementary material for: Double‐Stranded DNA Reduces dsRNA Degradation in the Saliva and Significantly Enhanced RNAi‐Mediated Gene Silencing in Halyomorpha halys
Source: Adv Biol (Weinh). 2025 Aug 17;9(9):e00698. doi: 10.1002/adbi.202400698 (PMC12447125; doi:10.1002/adbi.202400698)

ADVANCED BIOLOGY

Supporting Information

for *Adv. Biology*, DOI 10.1002/adbi.202400698

Double-Stranded DNA Reduces dsRNA Degradation in the Saliva and Significantly Enhanced RNAi-Mediated Gene Silencing in *Halyomorpha halys*

*Venkata Partha Sarathi Amineni, Georg Petschenka and Aline Koch**

Oneway Analysis of Corrected mortality rate By Treatment

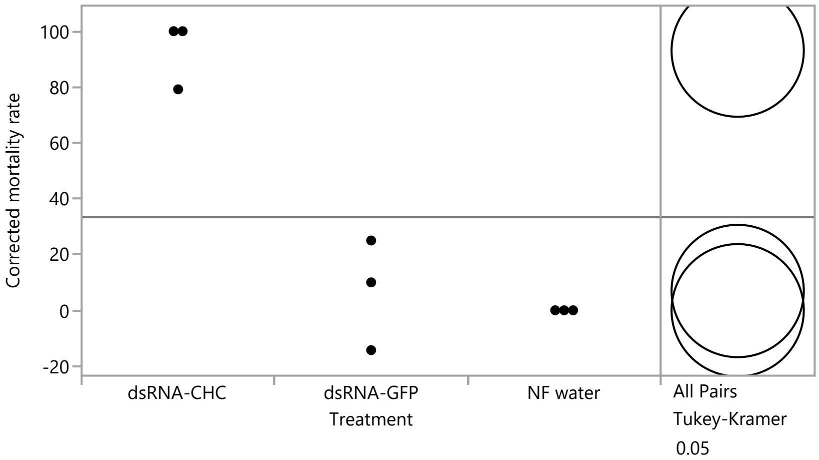

Means Comparisons

Comparisons for all pairs using Tukey-Kramer HSD

Confidence Quantile

q*	Alpha
3.06815	0.05

HSD Threshold Matrix

Abs(Dif)-HSD

	dsRNA-CHC	dsRNA-GFP	NF water
dsRNA-CHC	-33.538	52.612	59.517
dsRNA-GFP	52.612	-33.538	-26.634
NF water	59.517	-26.634	-33.538

Positive values show pairs of means that are significantly different.

Connecting Letters Report

Level			Mean	Std Error
dsRNA-CHC	A		93.056	7.7295
dsRNA-GFP		B	6.905	7.7295
NF water		B	0.000	7.7295

Levels not connected by same letter are significantly different.

Ordered Differences Report

Level	- Level	Difference	Std Err Dif	Lower CL	Upper CL	p-Value
dsRNA-CHC	NF water	93.05556	10.93113	59.5172	126.5939	0.0004*
dsRNA-CHC	dsRNA-GFP	86.15079	10.93113	52.6125	119.6891	0.0005*
dsRNA-GFP	NF water	6.90476	10.93113	-26.6336	40.4431	0.8089

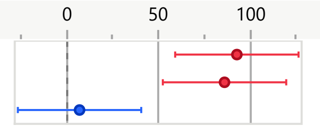

Supplement: Supplementary file 8 — Supporting Information [file ADBI-9-e00698-s003.pdf]
