# Supplementary material for: Double‐Stranded DNA Reduces dsRNA Degradation in the Saliva and Significantly Enhanced RNAi‐Mediated Gene Silencing in Halyomorpha halys
Source: Adv Biol (Weinh). 2025 Aug 17;9(9):e00698. doi: 10.1002/adbi.202400698 (PMC12447125; doi:10.1002/adbi.202400698)

ADVANCED BIOLOGY

Supporting Information

for *Adv. Biology*, DOI 10.1002/adbi.202400698

Double-Stranded DNA Reduces dsRNA Degradation in the Saliva and Significantly Enhanced RNAi-Mediated Gene Silencing in *Halyomorpha halys*

*Venkata Partha Sarathi Amineni, Georg Petschenka and Aline Koch**

## Generalized Linear Model Fit

Response: Dead
Modeling P(Dead=0)
Distribution: Binomial
Link: Logit
Estimation Method: Maximum Likelihood
Observations (or Sum Wgts) = 258

### Whole Model Test

| Model | -LogLikelihood | L-R ChiSquare | DF | Prob>ChiSq |
|---|---|---|---|---|
| Difference | 4.5716612 | 9.1433 | 7 | 0.2425 |
| Full | 127.790294 | | | |
| Reduced | 132.361956 | | | |

| Goodness Of Fit Statistic | ChiSquare | DF | Prob>ChiSq |
|---|---|---|---|
| Pearson | 258.3938 | 250 | 0.3442 |
| Deviance | 255.5806 | 250 | 0.3907 |

| AICc |
|---|
| 272.1589 |

### Effect Summary

| Source | Logworth | PValue |
|---|---|---|
| Repetition | 1.183 | 0.06563 |
| Treatment | 0.454 | 0.35161 |

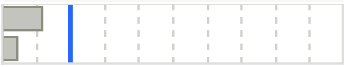

### Effect Tests

| Source | DF | L-R ChiSquare | Prob>ChiSq |
|---|---|---|---|
| Treatment | 6 | 6.678429 | 0.3516 |
| Repetition | 1 | 3.3891195 | 0.0656 |

### Parameter Estimates

| Term | Estimate | Std Error | L-R ChiSquare | Prob>ChiSq | Lower CL | Upper CL |
|---|---|---|---|---|---|---|
| Intercept | 1.9587465 | 0.3673193 | 35.375022 | <.0001* | 1.2692205 | 2.7152811 |
| Treatment[dsDNA-S] | -0.279829 | 0.3826715 | 0.5147344 | 0.4731 | -1.002003 | 0.5161431 |
| Treatment[dsRNA-CHC] | -0.276033 | 0.3534337 | 0.5901614 | 0.4424 | -0.947603 | 0.4503786 |
| Treatment[dsRNA-CHC+dsDNA-S] | -0.278153 | 0.4050021 | 0.4533541 | 0.5007 | -1.039667 | 0.5702877 |
| Treatment[dsRNA-GFP] | 0.4958112 | 0.4877362 | 1.1508931 | 0.2834 | -0.377156 | 1.5824723 |
| Treatment[dsRNA-GFP+dsDNA-S] | 0.6564913 | 0.4371326 | 2.5903151 | 0.1075 | -0.132885 | 1.6147329 |
| Treatment[Sucrose] | 0.1898058 | 0.3920209 | 0.2418918 | 0.6228 | -0.537636 | 1.0197955 |
| Repetition | -0.155495 | 0.0850652 | 3.3891195 | 0.0656 | -0.324832 | 0.0100181 |

## Generalized Linear Model Fit

### Studentized Deviance Residual by Predicted

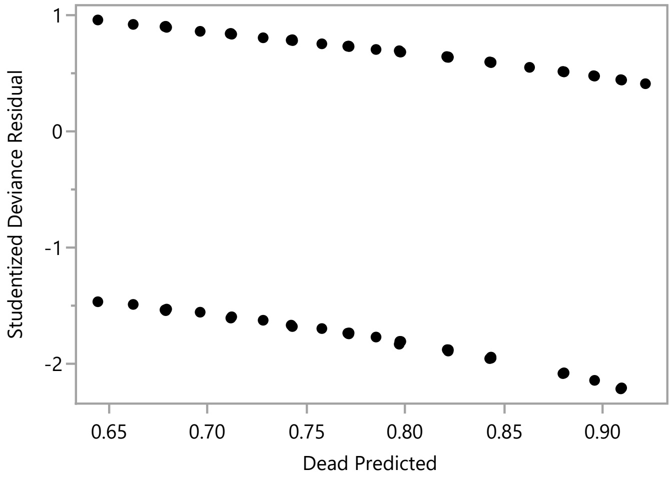

Supplement: Supplementary file 9 — Supporting Information [file ADBI-9-e00698-s002.pdf]
